# Single-cell transcriptomic dynamics of scallop heart reveals the heterogeneous response to heat stress

**DOI:** 10.1186/s12915-025-02210-1

**Published:** 2025-04-15

**Authors:** Danyang Wang, Na Liu, Xiangfu Kong, Xinghai Zhu, Yangfan Wang, Jingjie Hu, Zhenmin Bao

**Affiliations:** 1 MOE Key Laboratory of Marine Genetics and Breeding, College of Marine Life Sciences, Ocean University of China, Qingdao, China; 2 Fang Zongxi Center for Marine Evo Devo, Ocean University of China, Qingdao, China; 3 Laboratory of Tropical Marine Germplasm Resources and Breeding Engineering, Sanya Oceanographic Institution of the Ocean University of China (SOI-OUC), Sanya, China

**Keywords:** Open circulatory systems, Mollusks, Heart, Heat stress, ScRNA-seq, PLRP2

## Abstract

**Background:**

Animals with open circulatory systems are highly vulnerable to environmental temperature fluctuations, making them particularly threatened by global warming. However, research on the cellular heterogeneity of heart responses to elevated temperatures in animals with open circulatory systems remains limited.

**Results:**

Here, we conducted a comprehensive investigation of the morphology, metabolism and scRNA-seq of the heart in a molluscan model, *Argopecten irradians*, under heat stress. Our results unraveled that the severity of cardiac structure damage increased progressively with rising temperature, accompanied by widespread mitochondrial dysfunction and neurohumoral response. We identified two subpopulations within cardiomyocytes (CMs), including ventricular myocytes (VMs) and atrial myocytes (AMs), which exhibited specialized functional roles in response to thermal stress. Specifically, AMs enhanced cell–cell communications with the immune-like cells and fibroblasts to contribute to maintaining cardiac homeostasis under heat stress. Whereas, VMs displayed enhanced energy supply and differentiation potential to withstand thermal challenges. Furthermore, RNA interference targeting the most heat-responsive gene, *PLRP2-like*, resulted in a significant reduction in heat tolerance and triglyceride accumulation in scallops.

**Conclusions:**

Our study investigated the heterogeneous response of the scallop heart to high temperatures, revealing distinct response patterns between VMs and AMs. We further identified a key gene, *AiPLRP2-like*, which exhibits unique cellular localization patterns compared to its mammalian counterpart and may play a pivotal role in regulating cardiac thermotolerance in organisms with open circulatory systems. These findings provide novel insights into the theoretical framework and evolutionary adaptations of marine invertebrate hearts in response to environmental temperature fluctuations.

**Supplementary Information:**

The online version contains supplementary material available at 10.1186/s12915-025-02210-1.

## Background

Animals have two main types of circulatory systems. In all vertebrates and some invertebrates, the circulatory system is closed, meaning that blood is confined within blood vessels and does not flow freely within the body cavity [1]. In a closed circulatory system, blood circulates unidirectionally through the vessels, moving from the heart, along the systemic circulation route, and back to the heart. In contrast, most mollusks and arthropods have open circulatory systems. In an open circulatory system, blood (referred as hemolymph) is not contained within vessels but is pumped into a body cavity, where it mixes with interstitial fluid to facilitate gas and nutrient exchange with the organs. The open circulatory system lacks the complex vascular network and regulatory structures of a closed system, such as arteries, veins, capillaries, and sphincters, making it less effective at buffering temperature changes. Consequently, animals with open circulatory systems are particularly vulnerable to fluctuations in environmental temperature and are therefore at greater risk from global warming [2, 3]. The heart, as the circulatory system’s power source, plays a key role in maintaining blood pressure, regulating fluid balance, and supporting immune defense [4]. Under high temperatures, an increased heart rate boosts hemolymph flow for better heat dissipation. This also helps maintain fluid balance and osmotic pressure, particularly through kidney regulation, to counter dehydration [5]. As such, studying how the hearts of these animals respond to heat stress and how their heat tolerance can be enhanced is of critical importance.

Mollusca is the most species-rich phylum within Lophotrochozoa and one of the earliest bilaterians to appear in the fossil record [6]. The rich diversity, wide distribution from tropical to cold regions, and slow rate of evolution make mollusks an ideal model for studying the heat resistance of open circulatory systems [7]. Importantly, cardiac performance indices, particularly heart rate (HR) driven by cardiomyocytes, are widely recognized and employed as noninvasive indicators reflecting metabolic variations in mollusks in response to temperature alterations [8, 9]. These indices are also influenced by neurotransmitters [10]. According to previous surveys, most mollusks’ HR reached the peak followed by a sharp decline after heat stress, which is defined as the organism’s upper limit temperature. The temperature of the peak is called Arrhenius break temperature (ABT), which serves as a reliable indicator for quantifying the temperature tolerance of mollusks. ABT has been applied to various mollusks to distinguish the heat-tolerant and intolerant individuals, including scallop [11], mussel [12], oyster [13], and abalone [14]. Current research on heat tolerance traits primarily focuses on genomic and tissue-level expression analyses, such as whole-genome sequencing and cardiac transcriptome studies [15, 16]. However, the heart is a complex organ composed of multiple cell types, each potentially exhibiting distinct responses to heat stress. To comprehensively understand the mechanisms of heat tolerance and accurately identify key cell groups and regulatory genes, it is essential to investigate the heterogeneous responses of individual cell types with greater precision.

Recently, the rapid development of single-cell RNA sequencing (scRNA-seq) represents a powerful new tool for detecting comprehensive gene regulation at single-cell resolution in thousands of cells [17]. Using this method, numerous scRNA-seq datasets of vertebrate hearts were constructed (e.g., human [18], mouse [19], fish [20]) to identify cell types and unveil heterogeneity based on gene expression profiles, while, in the realm of marine invertebrates, only the heart lineage of sea squirt has been reported [21]. Additionally, the scRNA-seq has also been employed to study stress responses in marine animals, such as clarifying cell type-specific response to pathogen challenge in salmon liver [22], microplastic effects on tilapias gill [23], and copper exposure to oyster hemocytes [24].

As a representative among commercially important bivalves, the bay scallop's value stems not only from its rapid growth and high yield but also from its broad thermal resilience (from -1– 31℃), rendering it an ideal model organism for studying temperature tolerance mechanisms [25]. In the present study, we investigated alterations of HR, HR-related neurotransmitters, and energy metabolism enzyme activities in bay scallops during a temperature challenge experiment. Using scRNA-seq, we identified temperature-responsive genes and metabolism pathways in different cell clusters in response to heat exposure. Furthermore, siRNA-mediated RNAi was employed to knock down the most significantly expressed gene, *AiPLRP2-like*, to confirm its functional impact on cardiac performance and thermotolerance in bay scallop. To our knowledge, this study is the first to investigate the heterogeneous responses of the heart to high temperatures in invertebrates with open circulatory systems, providing the theoretical foundations of protecting marine invertebrates from the impacts of global warming.

## Results

### Structural injury and physiological metabolic alterations of scallop heart under heat stress

**Fig. 1:**
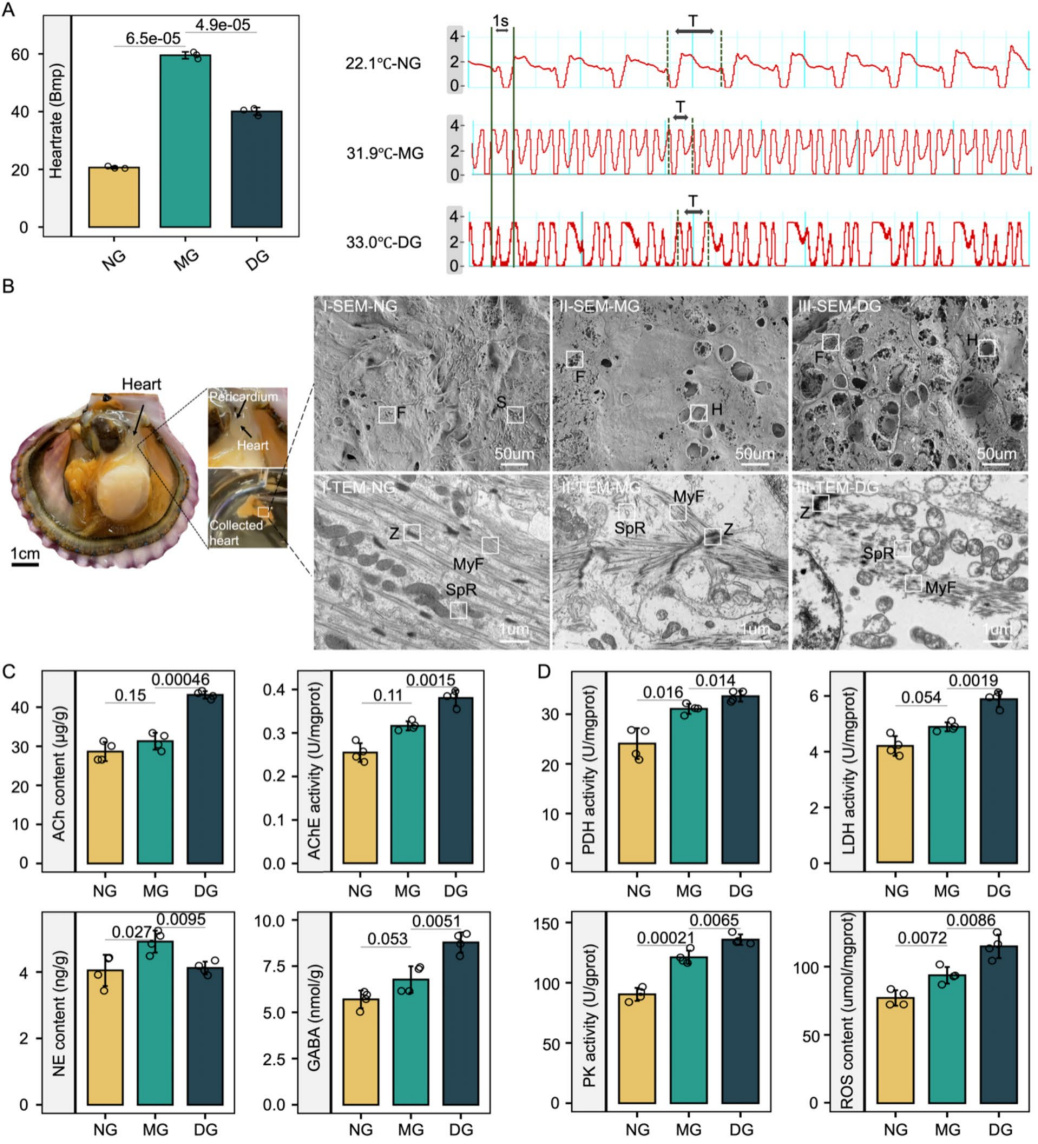
Impacts of heat stress on cardiac performance, morphology and metabolism of scallop hearts. **A** Heart rates (left) and representative cardiograms (right) of bay scallops under heat stress. **B** Scanning and Transmission electron microscopy (SEM and TEM) analysis. The characters F, H, S in SEM (I-III) stand for fissure, hole, and shrinking, respectively. The characters Z, MyF, and SpR in TEM (I-III) represent the cardiac structure Z-lines, Myofiber, and Sarcoplasmic reticulum, respectively. **C** In vivo contents of Norepinephrine (NE), Gamma-aminobutyric acid (GABA), acetylcholine (ACh), and activities of Acetylcholinesterase (AChE) in scallop hearts after exposure to heat stress (*n* = 4, mean ± SD). (D) The enzyme activities of PHD, LDH and PK, and ROS content of scallop hearts (*n* = 4, mean ± SD). The error bars represent SDs. The statistical significance was determined by a two-sided *t*-test and the *P*-values were shown above the plot

We collected scallop samples across three HR condition groups (Fig. 1A). The normal heart rate group (HR_nor_ group, NG), serving as the healthy control, was maintained at 22.1 ℃. The maximum heart rate group (HR_max_ group, MG), identified when cardiac activity peaked at approximately 60 bpm, was exposed to 31.9 ℃. The heart rate drop group (HR_drop_ group, DG), characterized by a sharp decrease in cardiac rate, was subjected to 33 ℃ water temperature. Notably, the samples used in this study exhibited consistency across different experiments (e.g., physiological indices, micro examination and scRNA-seq) under the same condition groups, as indicated by consistent heart rates (Additional file 1: Fig. S1). We first examined the morphological changes in the hearts of scallops during heat exposure using the SEM method. In comparison to the NG, the MG exhibited surface fissures with accompanying several large voids, while the DG displayed extensive voids and fragmentation. The TEM images of cardiac tissue revealed that both the MG and DG exhibited cardiac injury compared to the NG, with the DG showing more severe damage than the MG. The damage was characterized by blurred Z-lines, disordered muscle fibers, swollen sarcoplasmic reticulum, and mitochondrial swelling with sparse cristae (Fig. 1B).

We investigated the in vivo dynamics of three key neurotransmitters—ACh, NE, and GABA—along with AChE activity under heat stress. These neurotransmitters are crucial regulators of cardiac homeostasis in most vertebrates, mediated by the parasympathetic, sympathetic, and central nervous systems [26]. During the first stage of heat exposure, NE levels increased significantly by 1.21-fold (Fig. 1C), indicating an elevated heart rate, contractility, and conduction velocity in response to stress. ACh levels, AChE activity, and GABA levels showed only modest increases at this stage. However, during the second stage, these three metabolites exhibited substantial increases of 1.51-fold, 1.37-fold, and 1.54-fold, respectively, compared to MG (Fig. 1C), suggesting delayed negative feedback regulation that may help prevent arrhythmias. Concurrently, NE levels decreased by 19.63% compared to MG (Fig. 1C), underscoring the dynamic interplay between NE and ACh activity during heat exposure.

The dynamic changes in cardiac energy metabolism during heat stress were examined by assessing the levels of three energy metabolism-related enzymes (PDH, LDH, and PK), along with ROS. Nearly all of these metabolic indicators showed significant increases during both stages of heat exposure (Fig. 1D). Specifically, aerobic respiration-related indicators PDH and ROS increased by 1.29- and 1.22-fold during the first stage and by 1.08- and 1.23-fold during the second stage, respectively (Fig. 1D). These increases reflect the heart's response to elevated energy demands during heat stress, although excessive ROS may cause oxidative damage to cardiac cells. Additionally, anaerobic respiration-related indicators PK and LDH exhibited a sustained upward trend during both stages, increasing by 1.34- and 1.24-fold in the first stage and by 1.12- and 1.20-fold in the second stage, respectively (Fig. 1D). This suggests a compensatory mechanism to address the inability of aerobic respiration to fully meet energy demands promptly.

### Dynamic cellular landscape of scallop heart with scRNA-seq under heat exposure

**Fig. 2:**
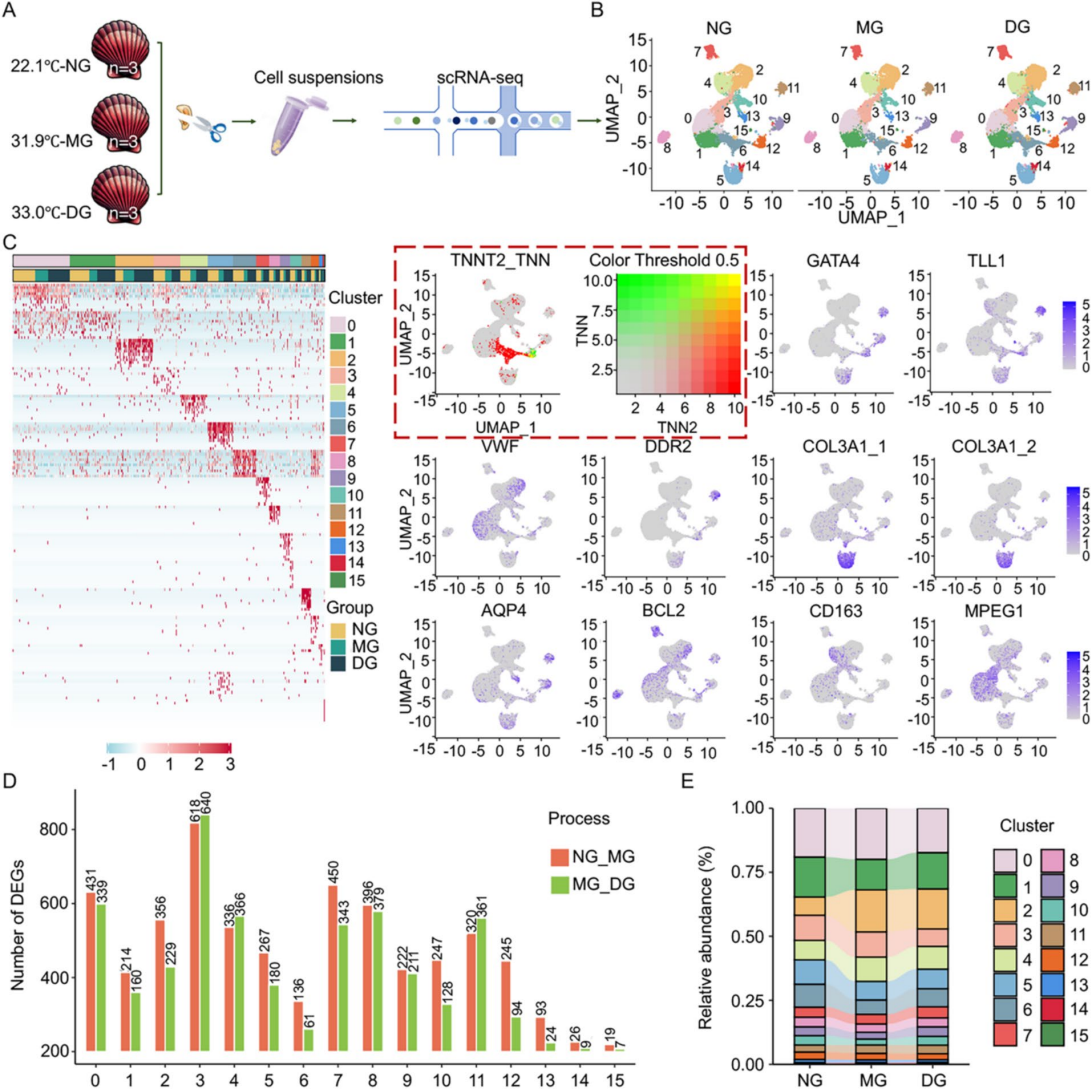
scRNA-seq analysis of heat untreated/treated scallop hearts. **A** Scallop heart collection from control and heat exposure groups, and sequenced with the microfluidics via 10 × Genomic platform. **B** UMAP plot analysis of 16 cell clusters in control (NG) and heat exposure (MG and DG) groups. **C** The heatmap (left) representing the top10 enriched genes among the 16 clusters. The cluster numbers correspond to the cluster shown in UMAP visualization. The umap (right) shows the expression patterns of the selected marker genes for each cell type and each dot in the graph represents a cell. The red rectangle emphasizes the umap of cardiomyocytes. **D** Differentially expressed genes (DEGs) and (**E**) relative abundance in 16 cell cluster of heart cells after heat exposure

To explore cell-specific responses, heart tissues collected from control and heat stress group (NG, MG, and DG) underwent scRNA-seq analysis (Fig. 2A). A total of 84,925 cells were isolated from three samples (Additional file 2: Table S1). After QC filtration, 50,048 high-quality cells (18,936 from NG, 10,735 from MG and 20,377 from DG) were obtained and used for subsequent cell cluster analysis. Additionally, the median number of the identified genes and mRNAs per cell were 1,152 and 3,272 for NG, 1,288 and 4,144 for MG, and 1,124 and 3,652 for DG, respectively (Additional file 2: Table S1).

The UMAP was employed to dimensionality reduction and unsupervised cell cluster analysis, identifying16 different cell clusters (0–15) with varied cells ranging from 30 to 3606 (Fig. 2B, Additional file 2: Table S2). Gene markers obtained from previous researches were used to define distinct cell subpopulations based on their specific expression in the corresponding cell type (Fig. 2C). Briefly, six cell types were identified in scallop heart tissue: cardiomyocytes (CMs, cluster 6 and 12), fibroblast cells (FCs, cluster 5, 11 and 14), cardiac progenitor cells (CPCs, cluster 9 and 15), endothelial cells (ECs, cluster 0, 1 and 2), astrocytes (ACs, cluster 13) and immune-like cells (ILCs), including macrophages (MPs, cluster 3, 4 and 10) and immune cells (ICs, 7, 8) (details in Additional file 2: Table S2). Notably, cardiomyocytes were further subdivided into ventricular (cluster 6) and atrial (cluster 12) subtypes based on the expression levels of marker genes [27] (*MYH9*, *MYO9B*, *POLR2A* and *SYT16*) (Additional file 1: Fig. S2). A heat map was constructed to display the top 10 enriched genes in each cluster, representing distinct molecular features across the 16 cell clusters (Fig. 2C). The differentially expressed genes (DEGs) in each cell cluster were also identified (*P*-value ≤ 0.05; log_2_FC ≥ 0.585) (Fig. 2D). Specifically, genes in MPs, ICs and ECs showed highly sensitive to heat stress, with the DEGs in these three accounting for over 50% of the total DEGs. Besides, cell types of FCs, CPCs, and CMs exhibited a moderate sensitivity, with 204, 120, and 190 DEGs (averaged by the number of clusters per cell type) identified during the first stage, and 183, 109, and 77 DEGs (averaged by the number of clusters per cell type) during the second stage. Additionally, the lowest number of DEGs was found in ACs, only 93 and 24 genes showing differential expression during two continuous stages.

Functional enrichment analysis showed that the DEGs (Additional file 1: Fig. S3A) of main cell groups in both heat exposure stages were primarily involved in cellular programs (e.g., ‘MAPK signaling pathway’, ‘mitophagy’, ‘necroptosis’, ‘apoptosis’ and ‘PI3K − Akt signaling pathway’) and responses to inflammation and immunity (e.g., ‘antigen processing and presentation’, ‘phagosome’ and ‘TNF signaling pathway’). In addition, during the first stage, DEGs were primarily associated with processes such as 'cardiac muscle contraction', 'thermogenesis', 'oxidative phosphorylation', and 'tight junctions'. Whereas, during the second stage, DEGs were mainly related to pathways including ‘focal adhesion’, ‘estrogen signaling pathway’ and ‘JAK − STAT signaling pathway’ (Additional file 1: Fig. S3B).

Furthermore, we explored the similarities and discrepancies in the response to heat exposure regulation among different subtypes of the same cell type. Venn diagram and KEGG analysis revealed significant differences among the subtypes, with a substantial proportion of subtype-specific DEGs (Additional file 1: Fig. S3-5). These findings suggest that distinct subtypes of the same cell type employ diverse molecular regulatory mechanisms to cope with thermal stimuli.

### Heat exposure triggered widespread mitophagy

**Fig. 3:**
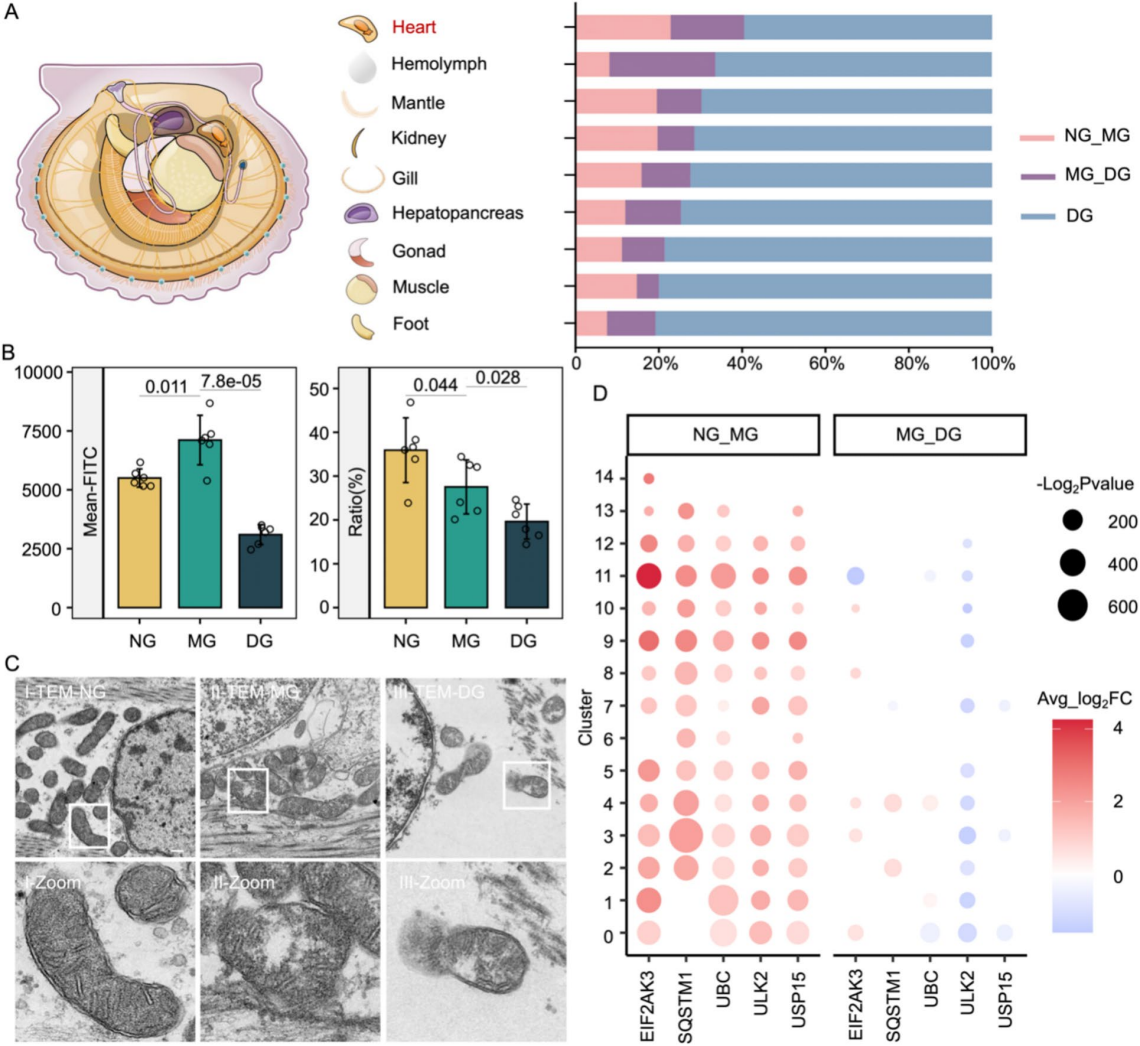
Energy changes in scallop heart after heat exposure. **A** ATP content variation in nine tissues (including heart, hemolymph, mantle, kidney, gill, hepatopancreas, gonad, muscle, and foot) of scallop in control (NG) and heat exposed groups (MG and DG). The pink and purple bars represent the percentage of energy consumption from NG to MG, and MG to DG out of the total energy consumption from NG. The blue bars represent the residual energy of ATP content. **B** The relative percentage of Mito-Tracker Green fluorescence levels (left), and the ratio of the red fluorescence to the green fluorescence that marked by JC-1 (right). **C** Representative mitochondrial status revealed by TEM analysis. The white rectangle framed and zoomed the representative mitochondria in NG, MG and DG. **D** The bubble shape represents the relative expression of mitophagy marker gene in 16 cell clusters from NG to MG and MG to DG. The significance and regulatory trend are illustrated by the size and color legend, respectively

The heart emerges as the tissue with the highest energy expenditure in response to heat stress among scallops. Across the entire heat exposure process, it consumes roughly 40% of the heart's energy reservoir (Fig. 3A). During the first heat exposure stage, the heightened energy demand prompts a sharp increase in the number of mitochondria (Fig. 3B). Concurrently, there is a pronounced surge in ROS level (Fig. 1D), leading to potential mitochondrial damage [28]. To maintain mitochondrial and cellular homeostasis, cells engage in a process known as mitophagy, wherein damaged or dysfunctional mitochondria are selectively packaged and degraded within the cell [29]. Our electron microscopy observations revealed that mitochondrial membranes swelled and the matrix exhibited cavitation during the first stage. Subsequently, during the second stage, mitochondrial membranes ruptured and crest fluid overflowed, resulting in severe damage (Fig. 3C). The continuous decline in membrane potential further indicated mitochondrial disorders (Fig. 3B) (details can be seen in Additional file 1: Fig. S6). Single-cell RNA analysis shows that the majority of cell types exhibited enhanced mitophagy during the first stage (Additional file 1: Fig. S7). It is worth noting that they invoke a common set of genes to facilitate mitophagy, comprising *EIF2AK3*, *SQSTM1*, *UBC*, *ULK2* and *USP15*. Among these genes, *ULK2* displayed a significant downregulation in most cell clusters after MG, whereas the expression levels of other genes experienced sporadic alterations or remained unchanged (Fig. 3D).

### Heterogeneity of energy metabolism in cardiomyocytes response to heat stress

**Fig. 4:**
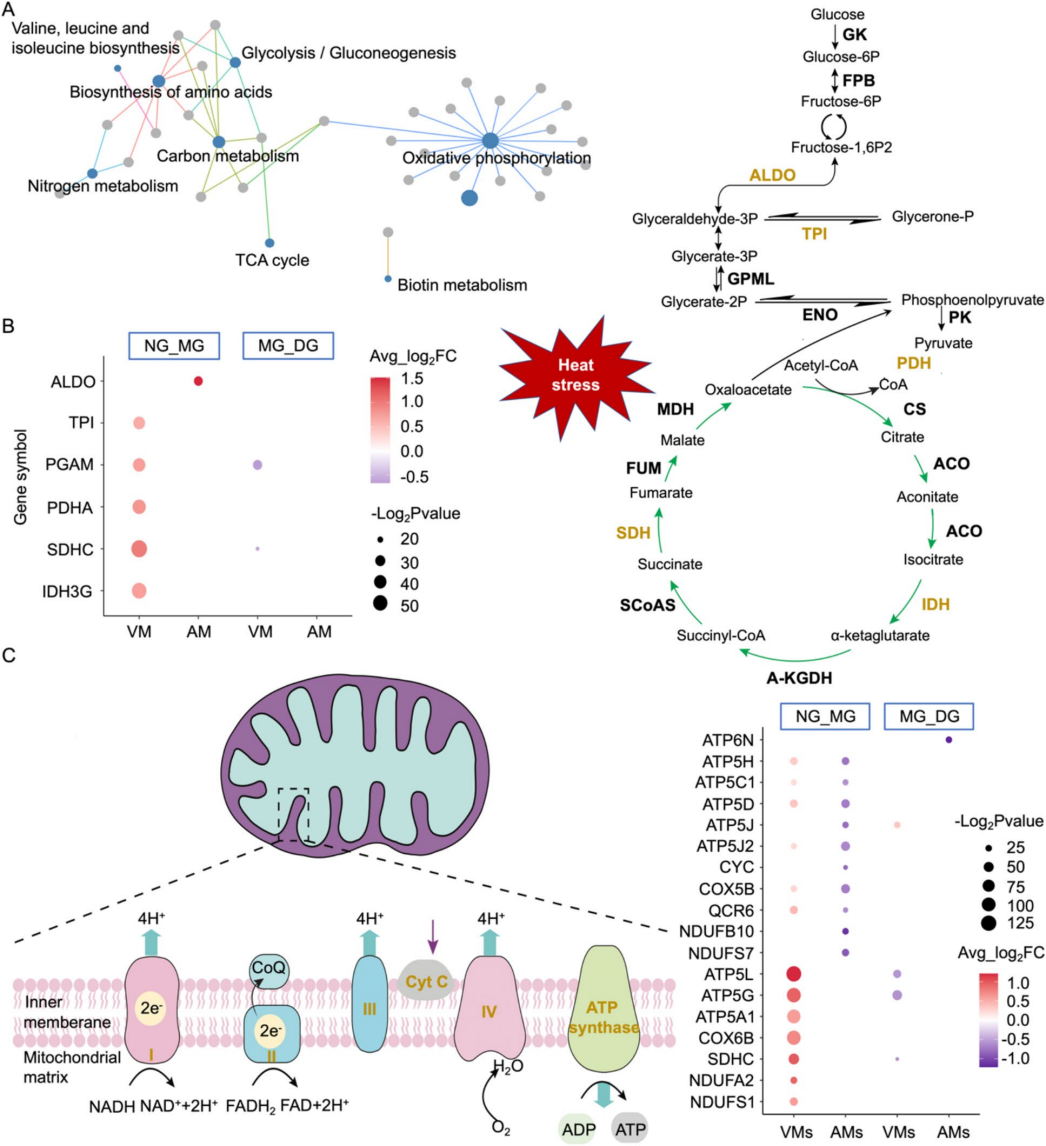
Ventricular myocytes (VMs) and atrial myocytes (AMs) exhibited distinct regulatory patterns in energy metabolism pathways under heat stress. **A** KEGG enrichment analysis of DEGs in VMs and AMs. **B** Predicted diagram of the energy metabolism pathway in VMs and AMs, including glycolysis, TCA cycle, and (**C**) oxidative phosphorylation process. The black bolded fonts indicate the key enzyme in the process and the yellow bolded fonts represent the genes displayed significant expression changes under heat stress. The size and color of bubble illustrate the significance and regulatory trend of pathway genes, respectively

The occurrence of mitochondrial autophagy during heat exposure hints at metabolic disturbances. Given their pivotal role in maintaining heart function, understanding how myocardial cells regulate their energy metabolism in response to heat stress is paramount for understanding the heat tolerance mechanisms of scallop hearts. After heat exposure, alterations in the energy metabolism of myocardial cells are primarily observed in glycolysis and oxidative phosphorylation pathways (Fig. 4A). Notably, the VMs and AMs display considerable disparities in the above two metabolic patterns. During the first heat exposure stage, the ventricle, serving as the primary regulator of glycolytic metabolism, generates rapid but limited amount of ATP energy by upregulating the *TPI*, *PGAM*, *PDHA*, *SDHC*, and *IDH3G* genes (Fig. 4B). Subsequently, during the second heat exposure stage, ventricular glycolytic metabolism remains sustained (Fig. 4B). Regarding the oxidative phosphorylation metabolic pathway, the ventricle and atria demonstrate contrasting coping strategies during the first stage. Specifically, VMs showed upregulation of gene expression encoding subunits of respiratory chain including Complex I (e.g., *NDUFA2* and *NDUFS*), Complex II (e.g., *SDHC*), Complex IV (e.g., *COX6B*) and Complex V (e.g., *ATP5L*, *ATP5G* and *ATP5A1*) (Fig. 4C). Whereas, AMs exhibited downregulation of genes involved in Complex I (e.g., *NDUFS7* and NDUFB10), Complex III (e.g., *QCR6*), Cytochrome c (e.g., *CYC*), Complex IV (e.g., *COX5B*) and Complex V (e.g., *ATP5J2* and *ATP5D*) (Fig. 4C). These expression patterns persist during the second stage, except for the downregulation of genes encoding some subunits in Complex V in both VMs and AMs (e.g., *ATP5L*, *ATP5G* in VMs and *ATP6N* in AMs) (Fig. 4C). Collectively, our findings underscore the distinct metabolic regulatory patterns of ventricular and atrial cardiomyocytes in response to heat exposure.

### Heat stress interfered the cell–cell communications between cardiomyocytes and other cell clusters

**Fig. 5:**
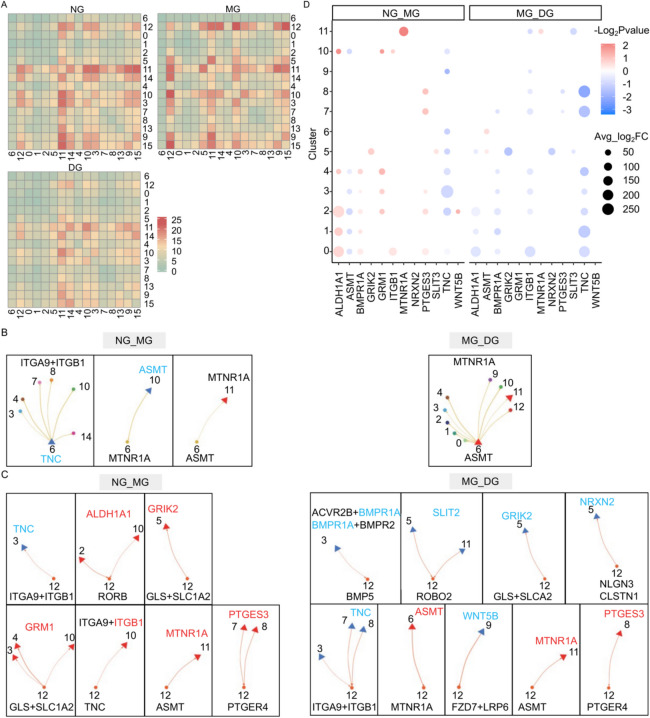
Cell–cell communications between cardiomyocytes (CMs) and other cell clusters. **A** Heatmap represents the quantity of ligand − receptor pairs, depicted by varying colors, among the 16 cell clusters in NG, MG and DG. **B**, **C** Significant alterations in ligand-receptor pairs between cardiomyocytes (6 for AMs and 12 for VMs) and other cell clusters were identified. Upregulated genes are denoted by red triangles, while downregulated genes are indicated by blue triangles. **D** Average log_2_FC of DEGs in (**B**, **C**) were shown. The significance and regulatory trend are illustrated by the size and color legend, respectively. Specifically, during the first stage, the heightened communication between AMs and ILCs primarily implicated signaling pathways related to retinoic acid (ALDH1A1-RORB), glutamate (GLS + SLC1A2-GRM1), and prostaglandins (PTGES3-PTGER4). During the second stage, the reduced intercellular communication between AMs and other cell populations predominantly involved BMP signaling (BMP5-BMPR1A + BMPR2/ACVR2B) with ILCs, neuroligin signaling (NLGN3-NRXN2) and calsyntenin signaling (CLSTN1-NRXN2) with FCs, and WNT signaling (WNT5B-FZD7 + LRP6) with CPCs, respectively. The intercellular communication between both AMs and VMs and FCs, mediated by melatonin signaling (ASMT-MTNR1A) exhibited a progressive increase during the two continuous stages

Cardiac fibroblasts and immune cells are key partners of cardiomyocytes and crucial regulators of cardiac homeostasis following injury in mammals [30, 31]. To explore how cellular communication between cardiomyocytes and other cell clusters responds to heat-induced heart injury, we analyzed ligand-receptor pairs among different cell clusters under three conditions. Heatmap comparisons showed significant changes in AMs compared to VMs (Fig. 5A). During the first stage, communication increased between AMs and FCs, ILCs, and CPCs (Fig. 5A). This increased interaction involved pathways related to retinoic acid, glutamate, and prostaglandins (Fig. 5C, D). In the second stage, intercellular communication was weakened (Fig. 5A), particularly between AMs and other populations, with a focus on BMP (Bone morphogenetic protein), neuroligin, and WNT signaling (Fig. 5C, D). Additionally, communication between AMs, VMs, and FCs, mediated by melatonin signaling, potentially implicated in wound healing and repair [32], showed a progressive increase across both stages (Fig. 5B-D). Overall, these results suggest that cardiac FCs and ILCs may play a preserved regulatory role in cardiac homeostasis during acute injury in scallops.

### Heat exposure induced transformation from cardiac progenitors to cardiomyocytes

**Fig. 6:**
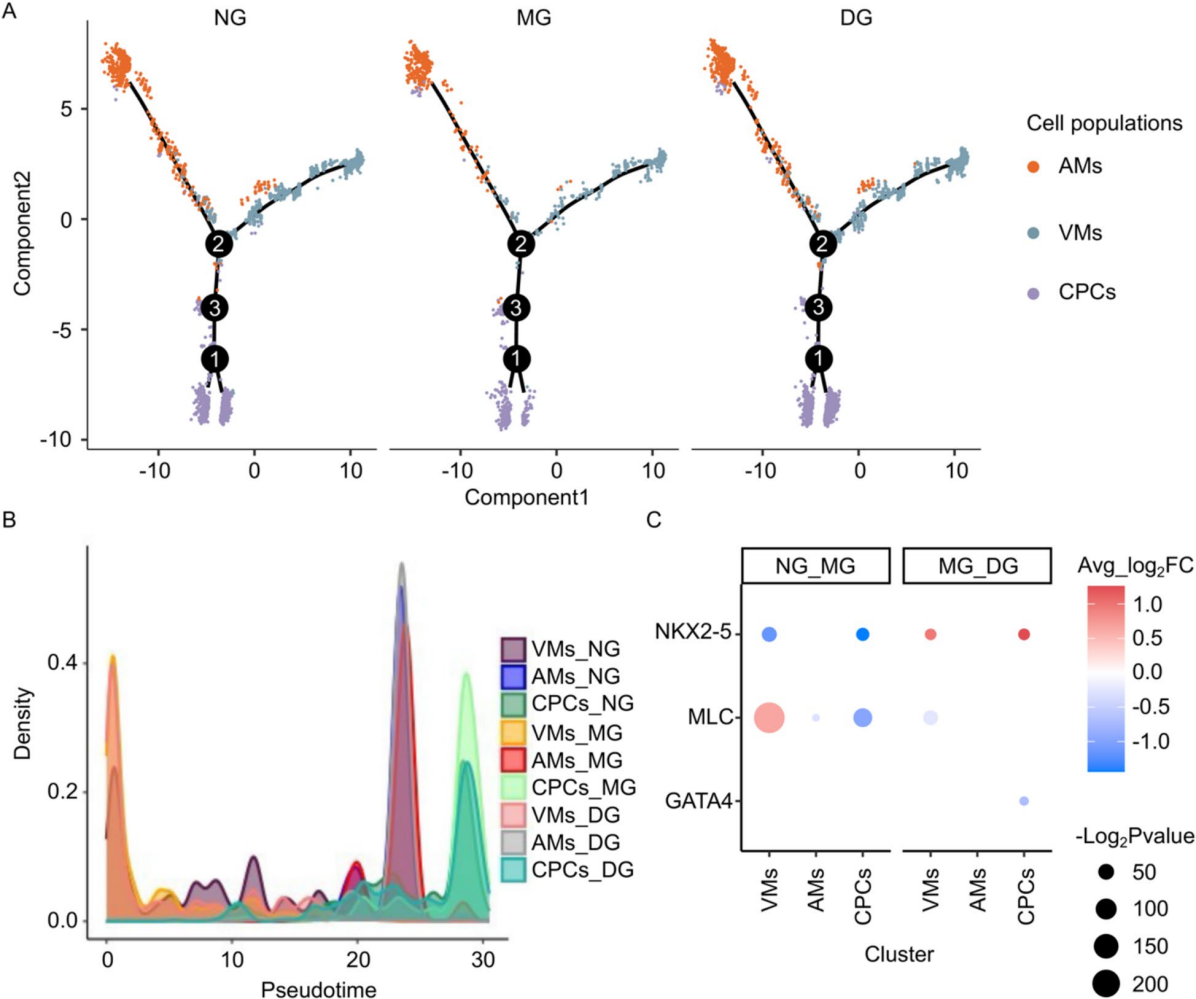
Pseudotime differentiation trajectories of CMs and CPCs. **A** Pseudotime ordering of AMs (cluster 12, orange), VMs (cluster 6, blue) and CPCs (cluster 9, purple) in NG, MG and DG. **B** The density distribution of AMs, VMs and CPCs over pseudotime in three conditions. **C** Average log_2_FC of *NKX2-5*, *MLC* and *GATA4* were shown. The significance and regulatory trend are illustrated by the size and color legend, respectively

Oxidative stress arises when the production of ROS surpasses the buffering capacity of antioxidant defense systems, leading to myocardial injury, ultimately culminating in cardiac dysfunction [33]. In our study, the observed mitochondrial collapse (Fig. 3B) and reduction in the proportion of cardiomyocytes (Fig. 2E) following heat exposure (from NG to MG) indicate myocardial damage in scallops. Investigating the regenerative potential of myocardial cells can enhance our understanding of bay scallops' ability to withstand heat exposure. To this end, we conducted pseudotime trajectory analysis to predict potential states of cell transition from cardiac progenitors to cardiomyocytes (Additional file 1: Fig. S8). Interestingly, we observed a notable trend where both the AMs and VMs tended to migrate away from CPCs during the first stage (Fig. 6A, B). However, during the second stage, this phenomenon in the AMs recovered, but persisted in the VMs (Fig. 6A, B). It is suggested by these findings that heat stress could prompt alterations in the population structure of myocardial cells, bolstering myocardial function to counter myocardial cell damage by augmenting the proportion of terminal myocardial cells. *NKX2-5*, *MLC* and *GATA4* are some of cardiomyocyte-selective genes that significantly contribute to myocardial regeneration [34–36]. In our study, the expression of *MLC* in VMs were significantly upregulated at MG compared to NG (average log_2_FC = 0.65). And the expression of *Nkx2-5* showed significant upregulation in both VMs and CPCs at DG compared to MG (average log_2_FC = 0.96 and 1.26, respectively) (Fig. 6C). These data indicate that VMs and CPCs exhibit stronger regenerative potential during the second stage, compared with AMs, which may align with the observed slight increase in cell proportion of VMs and CPCs during this period (from 5.6% and 2.6% to 7.0% and 3.7%, respectively) (Fig. 2E).

### Identification and expression pattern of AiPLRP2-like

**Fig. 7:**
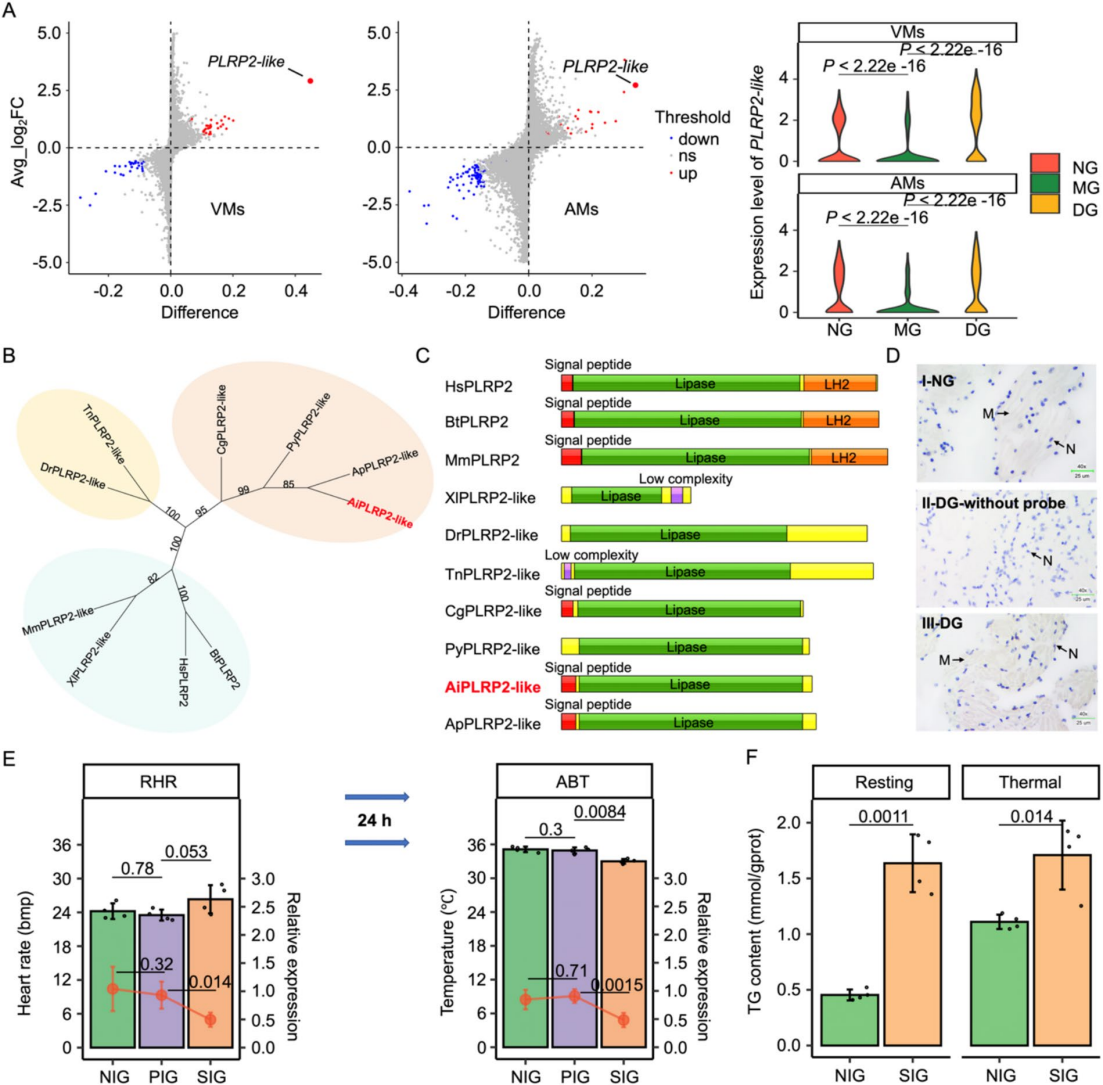
Identification and characterization of *AiPLRP2-like* in bay scallop. **A** Visualization of differentially expressed genes in VMs (cluster 6) and AMs (cluster 12) between MG and DG. **B**. The phylogenetic tree was constructed based on PLRP2s (-likes) sequences from bay scallop and other representative species (invertebrates and vertebrates shown with light red, light yellow and blue backgrounds, respectively. **C** Predicted protein domain architecture of AiPLRP2-like and other representative species PLPR2s (-likes) determined by SMART analysis. **D** In situ hybridization analysis of *AiPLRP2-like* in NG (I), DG-without probe (II), and DG (III). **E** Alterations in HR, ABT and *AiPLRP2-like* relative expression at the resting and thermal stage at 24 h post injection of siRNA- AiPLRP2-like and PBS. **F** The changes of TG content in NIG and SIG during resting and thermal, respectively. The vertical bars mentioned-above represent the mean ± SD (*n* = 4) and the statistical significance was determined by a two-sided *t*-test and the *P*-values were shown above the plot

Through scRNA-seq analysis, *AiPLRP2-like* was identified as the most significantly upregulated gene in both CM groups when the heart rate of scallop individuals sharply decreased, and it also showed a significant decrease during the first stage (Fig. 7A). These substantial changes suggest its potential role in cardiac function. To further explore whether *PLRP2-like* could be a key regulatory gene in open circulatory systems, we analyzed its conservation across different species. Phylogenetic analysis revealed that *PLRP2-like* proteins from marine invertebrates form a distinct cluster, separate from those in marine vertebrates and mammals (Fig. 7B). To gain deeper insights into the functional significance of *PLRP2-like*, we performed a structural analysis across both vertebrates and invertebrates. The results indicated that while both aquatic animals and mammals possess a Lipase domain, the LH2 domain present in mammals was absent in aquatic species (Fig. 7C), suggesting that although the function of *PLRP2* may be conserved, its cellular localization varies between aquatic species and mammals [37]. By comparing humans (Human Protein Atlas, proteinatlas.org), mice (Expression Atlas, ebi.ac.uk/gxa/home), scallops [15], and snails [27], we found that *PLRP2* is highly expressed only in the pancreas of vertebrates, while in mollusks, its homolog is highly expressed in both the heart and pancreas (Additional file 1: Fig. S9). DIG-labeled ISH further confirmed the widespread expression of *AiPLRP2-like* in the scallop heart, with stronger DAB staining observed in the DG compared to the NG, indicating higher mRNA expression (Fig. 7D). Taken together, these findings suggest that the absence of *PLRP2-like* localization domains in mollusks may allow *PLRP2-like* to regulate the heart in an open circulatory system.

### Effects of AiPLRP2-like knockdown and overexpression on cardiac performance and TG content

To further investigate the effect of *AiPLRP2-like* on heat tolerance in scallops, we performed an *AiPLRP2-like* knockdown experiment. Sixty scallops, with no significant differences in growth traits, were evenly divided into three groups: a non-injection group (NIG), a PBS (phosphate-buffered saline, 3 ×) injection group (PIG), and an siRNA injection group (SIG). The RNAi effects on resting heart rate (RHR) at 22.0 °C were then assessed. As shown in Fig. 7E, siRNA-mediated knockdown significantly suppressed the expression of *AiPLRP2-like*. The resting heart rate (RHR) of scallops in the SIG was slightly higher than that in both the NIG and PIG, 24 h post-injection. Additionally, the relative expression of *AiPLRP2-like* in SIG was significantly decreased by 52.50% and 46.81% compared to NIG and PIG, respectively. Moreover, the thermal indicator, ABT, was significantly reduced by approximately 2.13 ℃ and 1.93 ℃ in the SIG relative to the NIG and PIG. Taken together, these results suggest that *AiPLRP2-like* knockdown slightly accelerates RHR, but impairs thermal tolerance in bay scallops.

PLRP2 has been reported to break down triglyceride (TG) via its Lipase functional domain, thereby influencing cardiac function [38]. To determine whether *AiPLRP2-like* affects ABT by modulating TG content, we measured TG levels across different groups. The results showed a 2.60-fold increase in TG content in the SIG compared to the NIG at resting stage, rising from 0.45 ± 0.04 to 1.64 ± 0.22 mmol/gprot (Fig. 7F). Similarly, at the thermal stage, TG content in the SIG reached 1.55 mmol/gprot, representing a 0.40-fold increase compared to the NIG (Fig. 7F). Furthermore, we successfully overexpressed the target gene in H9C2 mouse cardiomyocyte cell lines (Additional file 1: Fig. S10A). TG content analysis of the collected cells revealed that the TG levels in cells overexpressing the *AiPLRP2-like* were significantly lower than those in the control group transfected with an empty vector (*n* = 3, *P* < 0.05) (Additional file 1: Fig. S10B). These findings indicate that the target gene indeed plays a functional role in break down triglyceride degradation.

## Discussion

Here, we present the first cardiac single-cell transcriptome in response to heat stress in the bay scallop, an animal with an open circulatory system. Our first notable finding is that heat exposure caused significant structural damage and energy metabolism alterations to the scallop heart. Microscopic examination revealed extensive surface fissures, voids, blurred Z-lines, and disordered myofibers in the DG group, suggesting a potential link between these structural deficiencies and the sharp decline in heart rate. This observation aligns with previous reports of myofiber damage associated with heart failure in mice [39]. As one of the most energy-demanding tissues, the heart is particularly vulnerable to alterations in energy metabolism, which can exacerbate conditions like muscle weakness, heart failure, and cardiac arrest [40]. Our findings indicate that the scallop heart exhibited the highest energy expenditure throughout the experiment. Further analysis of mitochondrial mass and mitophagy markers (Fig. 3D) revealed that elevated temperatures led to varying degrees of mitochondrial damage. Similar heat-stress-induced cardiac issues stemming from mitochondrial dysfunction have been reported in abalone [41], mussel [42], and snail [43]. These findings suggest that insufficient energy supply may be another contributing factor to the critical temperature-induced sharp decline in scallop heart rate.

Moreover, among the six identified cell types in the heart of *A. irrandians*, CMs are the primary constituents of heart cells and played an important role in heart contraction and relaxation [44]. To the best of our knowledge, this study represents the first attempt to identify subpopulations of CMs in scallop heart, and elucidate their heterogeneous responses to increasing temperatures. While CMs demonstrated a moderate response to high temperatures in bay scallop hearts, it is noteworthy that VMs and AMs exhibited distinct regulatory mechanisms in energy metabolism, likely attributable to their structural differentiation. The ventricular chamber of scallop showed similarity with vertebrates, being larger and stronger than atrial chamber [27], and thus consume more energy owing to temperature-induced acceleration of heartbeat, aligning with our findings of significant enhancement of energy-related pathways (oxidative phosphorylation and glycolysis) in VMs in MG group. By contrast, the AMs of scallop may exert the similar function to those of vertebrates AMs, primarily responsible for secretory signal transduction [45, 46], this was demonstrated by the current analysis of cell communications, which observed increased interactions between AMs and other cells clusters in stressed scallop heart.

Notably, a sharp heart rate decline was observed from MG to DG, which means the maximum metabolic rate of scallop fails to keep pace with routine metabolic rate as temperature increase [47, 48]. Identifying the key genes linked to high temperature-induced breaking point is thus pivotal for enhancing individual’s thermal tolerance. In present work, *AiPLRP2-like* was emerged as the most significantly up-regulated gene in both VMs (7.78-fold) and AMs (6.55-fold) per cell, and exemplified ubiquitous expressed in scallop heart via in situ hybridization. Such result might be different to conventional identity that *PLRP2* usually expressed in pancreas or intestine to hydrolysis and digestion of fat, cholesterol esters [49]. However, there are also some exceptions, as its broad range of hydrolysis substrate has also been reported to function in different tissues across various species [50]. For instance, *PLRP2* deletion impaired the homeostasis of undifferentiated spermatogonia and severely disrupted the lipid metabolism in mice sperm [51]. The *PLRP2* of insect brown planthopper that lacks the LH2-domain, has been proved to play a critical role in oocyte maturation and development, where its suppression significantly reduces egg hatch rate [52]. To validate the functions of *AiPLRP2-like*, gene knockdown was further adopted via RNA interference, which has been regarded as an efficient and powerful tool for elucidating the specific role of genes with uncertain roles to selectively silencing target genes [53]. Our result showed RHR in SIG was slightly higher than NIG and PIG (*P* = 0.1 and 0.053, respectively), while TG content was significantly elevated in SIG compared to NIG (*P* = 0.0011). This aligns with previous findings that *PLRP2* can specifically hydrolysis triglyceride into glycerol and fatty acid (FA), the reduced TG levels may associate with lower *AiPLRP2-like* mRNA expression [54]. HR-based thermal indicator ABT of scallop treated with or with *AiPLRP2-like-siRNA* injection to distinguish cardiac performance exposed to heat stress. The breaking point ABT of scallops in SIG is significantly decreased than those in NIG and PIG, with an average reduction of 2.03 ℃. The heart demands an increased energy supply to mitigate damage caused by thermal stress. However, reduced expression of *PLRP2* limits the availability of fatty acids for mitochondrial oxidation, leading to an insufficient energy supply and a subsequent decline in muscle contraction [49]. Additionally, triglyceride accumulation may exert toxic effects on the heart [55]. While several studies have explored the effects of rising temperatures on mariculture [56], the gene identified in our study provides new insights into the thermal tolerance mechanisms of marine invertebrates.

Neurotransmitters are widely recognized as another crucial factor impacting animal’s cardiac function and energy metabolism under stress condition, including heat stress [57, 58]. Our second notable finding revealed significant alterations of in vivo neurotransmitters (NE, ACh, and GABA) levels in scallop heart during heat experiment, exhibiting patterns of cardiac regulation similar to those observed in vertebrates. The release of NE led to faster heartbeats, while the increased secretion of ACh and GABA resulted a sharp decline in HR. This may be attributed to the acceleration of heartbeat leading to accelerate metabolic rate, stimulating glucose transport to meet the increased energy demands to cope with stress [59]. Conversely, sustained high-temperature stress imposes excessive workload on the heart, leading to a decrease in heart rate facilitated by significant releases of ACh and GABA, thus lessen cardiac functional disturbance [60]. The present outcome indicated the evolutionary conservation of neurotransmitters regulation pattern across vertebrates and invertebrates [61, 62].

Due to lack of adaptive immunity, the status of innate immunity is vital for mollusks countering the heat stress [63]. Our third notable finding is the successful identification of two types of immune-like cells (MPs and ICs) in bay scallop hearts, which are considered the most sensitive cell types in response to heat stress. Together, these cells accounted for approximately 50% of the differentially expressed genes (DEGs) throughout the entire experiment. The immune cells protect bivalve heart from pathogens infection and eliminate wounded cells upon external adverse environment, and such activated or perturbed immune response induced by high temperature were widely reported in bivalves [64]. Remarkably, the “Protein processing in endoplasmic reticulum” pathway, identified as the most significantly enriched pathway in KEGG analysis of ILCs and other four cell types, drives the upregulation of *HSP70/90*, *PERK*, *ATF4*, *ATF6B*, *TRAF2* and *BCL2*, thereby mitigating the protein aggregation when heart reached its maximum capacity in MG (Additional file 1: Fig. S11) [65]. By contrast, with the elevated temperature, two more pathways (*BAX1* and *JNK*) involved in cell death were evidently inhibited in ILCs and ECs once the heart is no longer bearing the heat stress. Besides, *GADD45* known for its pro-apoptotic effect on cells and ability to induce contractile dysfunction in cardiomyocytes [66], is significant down-regulated across all six cell types (except CPCs and ACs) in DG, along with myofibers disordered (Fig. 1B), implying that scallop may initiate self-protection through the suppression of apoptosis-related genes [67]. Moreover, the regular beating of heart relies on the normal release of calcium ions stored in the endoplasmic reticulum by cardiomyocytes [68]. Our study revealed that heat exposure resulted in swelling of sarcoplasmic reticulum and a notable increase in *CALR* and *CANX* expression across CMs, ECs, ILCs and FCs as detected by scRNA-seq data. These findings collectively suggest that high temperature may disrupt calcium hemostasis in ER, potentially causing a sharp decline in heartbeat.

Apart from activating the self-immune system to resist inflammation caused by elevated temperature, we also detected the slight increasing ratio of FCs and high enrichment of “ECM-receptor interaction” following with severely impairment of heart surface, which means more FCs may migrate toward the ripped site and produce extracellular matrix components to facilitate tissue regeneration [69, 70]. Moreover, ECs constitute the highest proportion (over 40% of total cell count) in heart tissue, a trait akin to that observed in vertebrates [71]. And their high temperature-induced DEGs mainly exert functions associated with “Cardiac muscle contraction” and “Nitrogen metabolism”. The neighboring CMs may be stimulated by changes in NO levels driven by ECs, thereby contributing to heart rate variation during heat exposure [72].

## Conclusions

In conclusion, our present work first evaluated cardiac performance, neurohumoral regulation and energy metabolism of bay scallop heart in response to critical temperature. Then, the scRNA-seq technique was firstly applied to characterize the cellular heterogeneous responses of scallop heart to increased temperature. According to the omics data, the notable enrichment of pathways such as "protein processing in the endoplasmic reticulum" and "mitophagy" across nearly all cell types under heat stress suggests a disruption in the regulation of protein and mitochondrial homeostasis. Importantly, we identified and characterized a novel *AiPLRP2-like* gene that showed the most significant upregulation in cardiomyocytes at the breaking temperature. Subsequent knockdown of *AiPLRP2-like* significantly weakened the thermal tolerance (-2.03 ℃ of ABT) of bay scallops. Our current study delves into the genetic and molecular biological underpinnings of heat tolerance, offering insights into the mechanisms behind the formation of the crucial heat tolerance indicator ABT in scallops.

## Methods

### Scallop culture and heat exposure experiment

Healthy one-year-old adult bay scallops (N > 500) were collected from artificial-rearing substrates in Huangdao, Qingdao (35°53′07" N, 120°8′42" E, Shandong Province, China) in December 2023 and transported to our laboratory (Ocean University of China, Qingdao, China) following standard procedure [73]. After removing encrusted organisms, scallops were acclimated with filtered and aerated seawater (22.03 ± 0.25 ℃ of temperature, 30.42 ± 0.47 ppt of salinity, 8.09 ± 0.04 of pH) for one week prior to experiment. Scallops were fed with 20 ml of concentrated algae fluid (*Nitzschia closterium*, ~ 2 × 10^9^ cells/mL) after partially replacing the water daily (1/2) during the rearing period.

In the heat exposure experiment, a total of 150 scallops (shell length at 58.29 ± 1.52 mm, mean ± SD) were transferred from the 22 ℃ seawater and randomly placed into three plastic basins filled with continuously aerated seawater, ensuring adequate oxygen supply. Based on the optimal growth temperature of 22 ℃ and the ABT of 32 ℃ of bay scallop, the temperature was increased from 22 ℃ to 34 ℃ with 0.2 ℃ per minute [74]. Scallop cardiac activity was monitored following the method described by Xing et al. [75]. In brief, the non-invasive infrared optical sensors CNY-70 were glued onto the shell surface close to cardiocoelom of the scallops, and signal variations were amplified, filtered and recorded via AMP03 and Powerlab (16/35, ADInstruments, Sydney, Australia). Due to the small size of scallop heart, three scallop hearts (one from each basin) were mixed as one sample to prepare heart cell suspension. Specifically, three scallops were randomly selected at 22 ℃ as the HR_nor_ group (NG) before the temperature increase. Subsequently, three more scallops were chosen for HR_max_ group (MG) when their heart rate reached 60 bmp (the peak HR observed in bay scallops), and three additional scallops were chosen for HR_drop_ group (DG) when a sharp drop in HR was detected. The real-time HR of each individual was continuously monitored and recorded during the whole experiment.

### Physiological indices in heart under heat exposure

To prepare samples for measurement, the scallop valve was opened, and heart was delicately dissected, and washed twice with sterile seawater to avoid hemolymph interference. The BCA (Bicinchoninic acid) protein assay kit (ML095490, MLBIO, Shanghai, China) was chosen to determine the protein concentrations of test samples and to standardize the following data values.

To investigate the impact of neurohumoral regulation on the heart under heat exposure, the in vivo contents of three neurotransmitters, including Norepinephrine (NE), Gamma-aminobutyric acid (GABA) and Acetylcholine (ACh) were determined in scallops at each group (*n* = 4, respectively) using corresponding commercial enzyme-linked immunosorbent assay (Elisa) kits (YJ120412, YJ970288, YJ095510, MLBIO, Shanghai, China) according to the manufacturer’s protocol. In brief, the test samples from four scallops from each experimental group were homogenized individually with PBS. The homogenates were centrifugated at 3000 g and 4 ℃ for 10 min. The specimens, standard samples, and HRP-labeled antibodies were sequentially added, followed by incubation (37 ℃, 60 min) and thorough washed for TMB staining. The in vivo content was measured in absorbance at 450 nm. The enzyme activity of Acetylcholinesterase (AChE) in scallop hearts (*n* = 4) after corresponding exposure was detected with an AChE activity kit (A024-1, Jiancheng, Nanjing, China). Briefly, the homogenates consisting of heart samples and PBS (weight:volume = 1:9) were centrifuged at 2500 rpm for 10 min, and the supernatant was then thoroughly mixed and incubation with working solution. Sample absorbance was measured at 412 nm and quantified by comparison with prepared standards.

To explore the energy metabolic change in the heart during heat stress, the in vivo contents of ATP (Adenosine triphosphate), Reactive oxygen species (ROS) (A095-1 and E004-1, Jiancheng, Nanjing, China) and the enzyme activities of LDH (Lactate dehydrogenase) and Phosphoenolpyruvate kinase (PK) (A020-2 and A076-1, Jiancheng, Nanjing, China), and Pyruvate dehydrogenase (PDH) (BC0385, Solarbio, Beijing, China) were detected at each group (*n* = 4, respectively) with corresponding commercial kits. Moreover, we also measured the ATP content in another eight different tissues (hemolymph, mantle, kidney, gill, hepatopancreas, gonad, muscle, and foot) in order to investigate the energy changes in different tissues of scallops in response to heat stress. The detection process was similar to the aforementioned AChE enzyme activity assay. Tissue homogenates were prepared and mixed with different working solutions. The results were measured at specific OD values and compared with standards.

### Microscopic examination of heart

Scanning and Transmission electron microscopy (SEM and TEM) were employed to assess the influence of heat stress on cardiac structure. The dissected hearts at each group were washed twice with sterile seawater to remove hemolymph and then promptly immersed in Electron microscope fixative (G1102, Servicebio, Wuhan, China) overnight at 4 ℃ to ensure optimal fixation of the morphology.

For SEM observation, the fixed hearts were dehydrated with alcohol, coated with gold after critical point-drying, and then the sample surface was examined through a SU8100 Scanning Electron Microscope (Hitachi, Tokyo, Japan). For TEM analysis, the fixed hearts were dehydrated with alcohol, embedded in epoxy resin, and sliced at a thickness of 60–80 nm. The image was examined in a HT7700 Transmission Electron Microscope (Hitachi, Tokyo, Japan) after stained with uranyl acetate and lead citrate.

### Evaluation of mitochondrial quality and quantity under heat exposure

Two commercial assay kit, Mito-Tracker Green and Mitochondrial membrane potential dye (MTG and JC-1, C1048 and C2003S, Beyotime Biotechnology, Nanjing, China) were used to evaluate the quantity and quality variation trend of mitochondria in scallop heart according to manufacturer’s construction (*n* = 6, respectively).

MTG, widely used as a fluorescent probe specific to mitochondria in live cells, can partially reflect mitochondrial quantity through average fluorescence intensity [76]. Briefly, 1000 *μ*L cell suspension (containing a total of 1 × 10^7^ cells) was extracted from one scallop heart from each group (NG, MG, and DG), 500 *μ*L of which were added to 500 *μ*L (20 nM) of Mito-Tracker Green solution, while the remaining 500 *μ*L was allocated for JC-1 analysis. After thorough mixing, the mixture was incubated with the fluorescence probes in the dark for 30 min. Subsequently, the samples were washed twice with 3 × PBS to avoid interference from working solution before proceeding with further analysis.

JC-1 kit is capable of distinguishing healthy (red) and the impaired (green) mitochondria through color differences [77]. Briefly, the remaining 500 *μ*L of cell suspension was mixed with 500 *μ*L of JC-1 working solution. Then, the samples were prepared for further analysis after washing twice with JC-1 buffer to remove working solution interference. Additionally, two sets of 500 *μ*L cell suspensions extracted from two scallop hearts in the NG group were with 1 *μ*L of carbonyl cyanide m-chlorophenyl hydrazone (CCCP, positive control) and none (blank control) for JC-1 analysis, respectively.

The prepared samples were analyzed using a BD FACSAria III flow cytometer system (Becton Dickinson, San Jose, California, USA), with at least 30,000 events recorded for each sample. Data analysis and visualization were performed via *Flowjo* software (v10.8.1) (Ashland, OR, USA).

### ScRNA-seq library preparation and sequencing

The heart tissues were digested in 1.5 mL centrifuges tubes containing 1 mL of type II collagenase (2 mg/mL) dissolved in 3 × PBS (the optimal osmotic pressure for scallop heart cells). After 30 min of dissociated on a shaker at room temperature, cell suspensions were filtered through a 40 μm nylon strainer. Following washes, centrifugation (600 g for 5 min), and resuspends with 3 × PBS to remove cell debris, the qualified cell suspension for library preparation exhibited over 90% viability (Trypan blue stained) and concentration of about 1000 cells/*μ*L. Importantly, the entire process was conducted on ice.

The beads, equipped with unique molecular identifiers (UMI) and cell barcodes, were loaded to near saturation, ensuring that each cell was associated with a bead in a Gel Beads-in-emulsion (GEM). Following exposure to cell lysis buffer, polyadenylated RNA molecules hybridized to the beads. The beads were subsequently collected into a single tube for reverse transcription. During cDNA synthesis, each cDNA molecule was labeled at the 5’ end (corresponding to the 3’ end of a messenger RNA transcript) with a UMI and cell label to denote its cell of origin. In summary, the 10 × beads underwent second-strand cDNA synthesis, adaptor ligation, and universal amplification. Sequencing libraries were meticulously crafted utilizing randomly interrupted whole-transcriptome amplification products to specifically amplify the 3’ end of transcripts associated with the cell barcode and unique molecular identifier (UMI). The subsequent procedures, encompassing library construction, adhered meticulously to the standard manufacturer’s protocol for Chromium Single Cell 3ʹ (v3.1). Quantification of the sequencing libraries was conducted using a High Sensitivity DNA Chip (Agilent) on a Bioanalyzer 2100 and the Qubit High Sensitivity DNA Assay (Thermo Fisher Scientific). Subsequently, the libraries underwent sequencing on the NovaSeq6000 (Illumina), yielding 150 bp paired-end reads.

### Initial processing of scRNA-seq data

Reads were aligned to the bay scallop genome (unpublished data) using *STAR* package [78] of *Cell Ranger* (v7.2.0) with default parameters. Next, Gene-Barcode matrices were generated for each individual sample by counting UMIs and filtering out non-cell associated barcodes. The scRNA-seq results were further analyzed using *Seurat* (v4.2.3) R package [79] for quality control and downstream analysis. The quality control analysis was performed with the following filtration: (1) the number of genes ≥ 200/cell; (2) UMI counts between 1000 and 20,000/cell; and (3) mitochondrial genes ≤ 20%. Then, the filtered data were normalized using the “LogNormalize” method with “scale.factor” = 10,000. We then scaled the data and performed principal component analysis (PCA). Unsupervised clustering was then performed using the FindNeighbors and FindClusters function. A resolution of 0.4 was selected as the clustering parameter to categorize clusters. We visualized the clusters on a 2D map produced with UMAP (Uniform manifold approximation and projection). For each cluster, the Wilcoxon Rank-Sum Test was applied to identify differentially expressed genes comparing the remaining clusters with the criteria set as follows: (1) *P*-value < 0.01; (2) log_2_FC ≥ 0.25; and (3) the percentage of cells in which the gene was detected in a specific cluster > 25%.

### Identification of DEGs and enrichment analysis

For individual cell types, differential expression comparing only two groups by condition was performed using the FindMarkers function of *Seurat* with the following criteria: (1) |log_2_FC|≥ 0.585; (2) *P*-value ≤ 0.05; and (3) the percentage of cells in which the gene was detected in at least one condition > 25%.

Functional-enrichment analysis KEGG were performed to identify which DEGs were significantly enriched in KEGG terms and metabolic pathways at Bonferroni-corrected *P*-value ≤ 0.05 compared with the whole-transcriptome background.

### Single-cell trajectory analysis

The trajectories of myocardial-related cell types (cluster 6, 9, and 12) were inferred using the *Monocle* (v3) R package [80]. In essence, Monocle orders individual cells along a pseudotime trajectory, capturing their asynchronous progression in biological processes like cell differentiation. This approach allows for the reconstruction of developmental trajectories and provides insights into the dynamic processes occurring in distinct cell clusters.

### Cell–cell interaction analysis

The identification of ligand–receptor pairs between two cell types was conducted using *CellphoneDB* (v2.0) software [81], relying on the expression of a receptor in one cell type and a ligand in another. Statistical enrichment was assessed through a permutation-based approach. The gene library primarily drew references from humans.

### Identification and characterization analysis of AiPLRP2-like

First, we initially fetched the candidate sequence following the methodology delineated in our previous study [82]. The protein sequences corresponding to PLRP2 (-likes) from both vertebrates and invertebrates (Additional file 2: Table S3) were sourced from the National Center for Biotechnology Information (http://www.ncbi.nlm.nih.gov). These sequences were aligned using the *ClustalW2* algorithm to facilitate downstream phylogenetic analysis. Phylogenetic tree was subsequently generated with *MEGAX* software (v10.1.8) [83] employing the maximum-likelihood approach, supported by 1,000 bootstrap replications to ensure statistical robustness.

In situ hybridization (ISH) was performed to determine the location of *AiPLRP2-like* mRNA expressed in scallop heart. For the probe, the cDNA extraction of heart samples was conducted following the procedure in our previous work [82]. Based on full-length cDNA sequence of *AiPLRP2-like*, 410 bp fragments were amplified using the gene-specific primers F and R7 (Table 1), and purified by a magnetic bead-based purification method for subsequent in vitro transcription. Digoxigenin-labeled sense and anti-sense probes were synthesized using a DIG RNA Labeling Kit (T7, Roche, Mannheim, Germany). Fixed heart tissues sections (~ 4 μm) from NG, MG and DG group were deparaffinized, incubated with prehybridization solution, and hybridized with the probe. Then, the tissue sections were visualized using 3,3’-diaminobenzidine (DAB), and images were displayed via a confocal laser scanning.

**Table 1:** Sequences of primers used in this study

Primer	Nucleotide sequence	Note
EF1a	F: 5’- ACTGGAACCTCCCAAGCCGAT-3’	Reference gene
	R: 5’- TTTACACCAAGCGTGTAGGCGAG-3’	
PLRP2-like	F: 5’- GTGCTTATGAGGTCTGGGTTC-3’	RT-qPCR
	R: 5’- GACAGACTGCTGGTACATCTTAC-3’	
siRNA	F: 5’- CUGUUUACAGAGUCAUUACTT-3’	Interference
	R: 5’- GUAAUGACUCUGUAAACAGTT-3’	
Probe	F: 5’- CCATCTGACGCTTTGTTTG-3’	In situ hybridization
	R7: 5’- **TACGACTCACTATAGGG**GAGTG ACCGGAAGTAGTTAAG-3’	
PLRP2-like	F: 5’- AGTCCGGACTCAGATCTCGAGGC AAGTAGACGGATCCCGG-3’ R7: 5’- GGATCCCGGGCCCGCGGTACCC TGACAGTAGGGAGAGTGACCG-3’	Overexpression

The bold characters in primers R7 indicate T7 promoter sequence

### AiPLRP2-like knockdown effects on cardiac performance

To confirm the role of *AiPLRP2-like* on cardiac performance, in vivo knockdown of *AiPLRP2-like* was conducted via siRNA-executed RNAi. Primers was designed using *siDirect* (v2.0) online tool and synthesized by Sangon BioTech (Shanghai, China). Over 500 healthy bay scallops were randomly collected from artificial scallop-rearing substrates in Huangdao, Qingdao (35°52′58" N, 120°9′30" E, Shandong Province, China), and acclimated for one week in filtered and aerated seawater (22.15 ± 0.24 °C of temperature, 8.12 ± 0.18 of pH, 30.10 ± 0.25 ppt of salinity) for one-week acclimation with half of the seawater replaced daily.

Two key cardiac indices, RHR and the thermal tolerance indicator ABT, were measured 24 h after *AiPLRP2-like* siRNA injection. Sixty scallops, with no significant difference in growth traits, were evenly divided into three groups, the none-injection group (NIG), PBS (Phosphate buffer saline, 3 x)-injection group (PIG), and siRNA-injection group (SIG) to assess RNAi effects on RHR at 22.0 ℃. Each scallop in the SIG was conducted with 20 μl (20 μM)* AiPLRP2-like* specific siRNA injection into the adductor muscle, while the PIG was injected with an equivalent volume of PBS (3 x), and the NIG served as the control. Four samples from each group were randomly selected to detect their heart rate variation 24 h post-injection. Additionally, another four scallops per group were selected to determine ABT, assessing the impact of *AiPLRP2-like* siRNA on thermotolerance. Following heart rate recording of each scallop, RNA isolation, cDNA synthesis, and *AiPLRP2-like* expression were performed. The full-length coding sequence of *AiPLRP2-like* is available in GenBank (PQ565843), and primers for siRNA synthesis and qRT–PCR are provided in Table 1.

### AiPLRP2-like knockdown effects on triglyceride content

We quantified triglyceride level using a commercial kit (A110-1, Jiancheng, Nanjing, China). Four per scallop hearts from the NIG and SIG at resting and thermal stages were collected and processed following the manufacture’s instruction. The principle primarily relies on the oxidation of triglyceride degradation products by oxidase enzymes, resulting in the formation of red quinonoid compounds. The intensity of the color is proportional to the TG content.

### AiPLRP2-like overexpression effects on triglyceride content

The cDNA extraction of bay scallop was conducted following the procedure in our previous work [82] and the open reading frames (ORFs) of *AiPLRP2-like* was identified by comparing to the bay scallop genome (unpublished data). The sense and antisense primers (Table 1) for PCR were marked respectively with XhoI and KpnI restriction sites to obtain fragments used for vector reconstruction supported by ClonExpress II One Step Cloning Kit (C112, Vazyme, Nanjing, China). The constructed pEGFP-C1-AiPLRP2-like plasmid was transformed into DH5α cells (Biotechnology Technologies, China) to select recombinants containing the correct fragments, which were further confirmed by sequencing verification (Sangon Biotech Co., Ltd.). H9C2 cells (Procell, Wuhan, China) were cultured in a 6-well plate under conditions of 37 °C and 5% CO₂, using 1 mL of DMEM medium (11,995, Solarbio, China) supplemented with 10% FPS (SV30208, Hyclone, Australia). When the cell density reached approximately 70%, the medium was then replaced with serum-free medium for starvation treatment. The cells were then transfected with either pEGFP-C1-AiPLRP2-like plasmid (2 ng per well, *n* = 3) or the pEGFP-C1 plasmid (2 ng per well, *n* = 3) using Lopofectamine 3000 (Thermo Fisher Scientific). After 6 h, the medium was replaced with complete culture medium and the cells were cultured for an additional 24 h. To verify the success of transfection, fluorescence microscopy was used to observe the green fluorescent protein tag. To assess the alteration of triglyceride content in cells, H9C2 cells were initially rinsed three time with 1 × PBS (pH 7.5), and then we quantified triglyceride level using a commercial kit (A110-1, Jiancheng, Nanjing, China).

### Statistical analysis

To analyze physiological indices (e.g., Heart rate, ATP content, NE activity) changes and mitochondrial quality and quantity evaluation in bay scallop, significant difference in the above-mentioned indicators among control (NG), HR_max_ group (MG), and HR_drop_ group (DG) were determined using two-side t-test embed in *Ggpubr* (v0.4.0) R package [84]. For qRT–PCR analysis, the relative expression levels of genes were calculated according to the comparative 2^−ΔΔCt^ method.

## Supplementary Information

Additional file 1: Fig. S1. The histogram of heart rate of bay scallop in groups. Fig. S2. Expression profile of cardiac markers to categorize ventricle and atria in bay scallop. Fig. S3. Venn diagram and KEGG analysis of DEGs in five main cell types. Fig. S4. KEGG analysis of DEGs in CMs and ECs. Fig. S5. KEGG analysis of DEGs in FCs and ILCs. Fig. S6. The raw data of flow cytometry results from MTG and JC-1 analysis. Fig. S7. KEGG analysis of up- and down-regulated DEGs in six cell types. Fig. S8. Pseudotime ordering of cells, including AMs (cluster 12), VMs (cluster 6), and CPCs (cluster 9) in three groups. Fig. S9. The expression profile of *PLRP2s(-likes)* in representative vertebrates (human and mouse) and invertebrates (scallop and snail). Fig. S10. Fluorescence microscopy of pEGFP-C1-transfected cells and triglyceride levels across experimental groups. Fig. S11. The expression profile of DEGs in “Protein processing in the endoplasmic reticulum” pathway.

Additional file 2: Table S1. The statistical data from three scRNA-seq databases. Table S2. The proportion of cell types and their corresponding identity markers. Table S3. PLRP2 protein sequences information used in this study.

## Data Availability

The single-cell transcriptome data that support the findings of this study have been deposited in the NCBI Sequence Read Archive with accession PRJNA1187280, the link is list below: https://www.ncbi.nlm.nih.gov/sra/?term=PRJNA1187280.

## Abbreviations

ABT: Arrhenius break temperature
AChE: Acetylcholinesterase
Ach: Acetylcholine
ACs: Astrocytes
AMs: Atrial myocytes
ATP: Adenosine triphosphate
BCA: Bicinchoninic acid
BMP: Bone morphogenetic protein
CCCP: Carbonyl cyanide m-chlorophenyl hydrazone
CMs: Cardiomyocytes
CPCs: Cardiac progenitor cells
DAB: 3,3’-Diaminobenzidine
DEGs: Differentially expressed genes
DG: The heart rate drop group
DIG-labeled ISH: Digoxigenin-labeled in situ hybridization
ECs: Endothelial cells
FA: Fatty acid
FCs: Fibroblast cells
GABA: Gamma-aminobutyric acid
GEM: Gel Beads-in-emulsion
HR: Heart rate
ICs: Immune cells
ILCs: Immune-like cells
JC-1: Mitochondrial membrane potential dye
KEGG: Kyoto encyclopedia of genes and genomes
LDH: Lactate dehydrogenase
MG: The maximum heart rate group
MPs: Macrophages
MTG: Mito-tracker green
NG: The normal heart rate group
NIG: Non-injection group
NE: Norepinephrine
ORFs: Open reading frames
PCA: Principal component analysis
PDH: Pyruvate dehydrogenase
PIG: PBS (phosphate-buffered saline, 3 ×) injection group
PLRP2-like: Pancreatic lipase-related protein 2-like
PK: Phosphoenolpyruvate kinase
RHR: Resting heart rate
RNAi: RNA interference
ROS: Reactive oxygen species
scRNA-seq: Single-cell RNA sequencing
SEM: Scanning electron microscopy
SIG: SiRNA injection group
TG: Triglyceride
TEM: Transmission electron microscopy
UMAP: Uniform manifold approximation and projection
UMI: Unique molecular identifiers
VMs: Ventricular myocytes

## References

[1] Reiber CL, McGaw IJ. A review of the “open” and “closed” circulatory systems: new terminology for complex invertebrate circulatory systems in light of current findings. Int J Zool. 2009;1:301284.

[2] Scanes E, Byrne M. Warming and hypoxia threaten a valuable scallop fishery: a warning for commercial bivalve ventures in climate change hotspots. Glob Change Biol. 2023;29(8):2043–5. 10.1111/gcb.16606 36655296

[3] Soon TK, Zheng H. Climate change and bivalve mass mortality in temperate regions. Rev Environ Contam Toxicol. 2020;251:109–29. 31289937 10.1007/398_2019_31

[4] Simoes-Costa MS, Vasconcelos M, Sampaio AC, Cravo RM, Linhares VL, Hochgreb T, et al. The evolutionary origin of cardiac chambers. Dev Biol. 2005;277(1):1–15. 15572135 10.1016/j.ydbio.2004.09.026

[5] Wilson TE, Crandall CG. Effect of thermal stress on cardiac function. Exerc Sport Sci Rev. 2011;39(1):12–7. 21088607 10.1097/JES.0b013e318201eed6 PMC3076691

[6] Budd GE. The earliest fossil record of the animals and its significance. Philos Trans R Soc Lond B Biol Sci. 2008;363(1496):1425–34. 18192192 10.1098/rstb.2007.2232 PMC2614223

[7] McMahon BR, Wilkens JL, Smith PJ. Invertebrate circulatory systems. Compr Physiol. 2011;931–1008.

[8] Eymann C, Götze S, Bock C, Guderley H, Knoll AH, Lannig G, et al. Thermal performance of the European flat oyster, Ostrea edulis (Linnaeus, 1758)—explaining ecological findings under climate change. Mar Biol. 2020;167:1–15.

[9] Liao ML, Li GY, Wang J, Marshall DJ, Hui TY, Ma SY, et al. Physiological determinants of biogeography: the importance of metabolic depression to heat tolerance. Glob Change Biol. 2021;27(11):2561–79. 10.1111/gcb.15578 33666308

[10] Tang Y, Du X, Sun S, Shi W, Han Y, Zhou W, et al. Circadian rhythm and neurotransmitters are potential pathways through which ocean acidification and warming affect the metabolism of thick-shell mussels. Environ Sci Technol. 2022;56(7):4324–35. 35293730 10.1021/acs.est.1c06735

[11] Xing Q, Li Y, Guo H, Yu Q, Huang X, Wang S, et al. Cardiac performance: a thermal tolerance indicator in scallops. Mar Biol. 2016;163:1–9.

[12] Braby CE, Somero GN. Following the heart: temperature and salinity effects on heart rate in native and invasive species of blue mussels (genus Mytilus). J Exp Biol. 2006;209(13):2554–66. 16788038 10.1242/jeb.02259

[13] Jiang G, Zhou J, Cheng G, Meng L, Chi Y, Xu C, et al. Examination of survival, physiological parameters and immune response in relation to the thermo-resistant heterosis of hybrid oysters derived from Crassostrea gigas and C. angulata. Aquaculture. 2022;559:738454.

[14] Chen N, Luo X, Gu Y, Han G, Dong Y, You W, et al. Assessment of the thermal tolerance of abalone based on cardiac performance in Haliotis discus hannai, H. gigantea and their interspecific hybrid. Aquaculture. 2016;465:258–64.

[15] Zhu X, Zhang J, Li M, Hou X, Liu A, Dong X, et al. Cardiac performance and heart gene network provide dynamic responses of bay scallop Argopecten irradians irradians exposure to marine heatwaves. Sci Total Environ. 2023;882:163594. 37094688 10.1016/j.scitotenv.2023.163594

[16] Zhu X, Liu P, Hou X, Zhang J, Lv J, Lu W, et al. Genome-wide association study reveals PC4 as the candidate gene for thermal tolerance in bay scallop (Argopecten irradians irradians). Front Genet. 2021;12:650045. 34349776 10.3389/fgene.2021.650045 PMC8328476

[17] Kharchenko PV. The triumphs and limitations of computational methods for scRNA-seq. Nat Methods. 2021;18(7):723–32. 34155396 10.1038/s41592-021-01171-x

[18] Koenig AL, Shchukina I, Amrute J, Andhey PS, Zaitsev K, Lai L, et al. Single-cell transcriptomics reveals cell-type-specific diversification in human heart failure. Nat Cardiovasc Res. 2022;1(3):263–80. 35959412 10.1038/s44161-022-00028-6 PMC9364913

[19] Skelly DA, Squiers GT, McLellan MA, Bolisetty MT, Robson P, Rosenthal NA, et al. Single-cell transcriptional profiling reveals cellular diversity and intercommunication in the mouse heart. Cell Rep. 2018;22(3):600–10. 29346760 10.1016/j.celrep.2017.12.072

[20] Cao J, Navis A, Cox BD, Dickson AL, Gemberling M, Karra R, et al. Single epicardial cell transcriptome sequencing identifies Caveolin 1 as an essential factor in zebrafish heart regeneration. Development. 2016;143(2):232–43. 26657776 10.1242/dev.130534 PMC4725347

[21] Wang W, Niu X, Stuart T, Jullian E, Mauck WM III, Kelly RG, et al. A single-cell transcriptional roadmap for cardiopharyngeal fate diversification. Nat Cell Biol. 2019;21(6):674–86. 31160712 10.1038/s41556-019-0336-z PMC7491489

[22] Taylor RS, Ruiz Daniels R, Dobie R, Naseer S, Clark TC, Henderson NC, et al. Single cell transcriptomics of Atlantic salmon (Salmo salar L.) liver reveals cellular heterogeneity and immunological responses to challenge by Aeromonas salmonicida. Front Immunol. 2022;13:984799. 36091005 10.3389/fimmu.2022.984799 PMC9450062

[23] Zheng S, Wang WX. Single-cell RNA sequencing profiling cellular heterogeneity and specific responses of fish gills to microplastics and nanoplastics. Environ Sci Technol. 2024;58(13):5974–86. 38512049 10.1021/acs.est.3c10338

[24] Meng J, Wang WX. Highly sensitive and specific responses of oyster hemocytes to copper exposure: single-cell transcriptomic analysis of different cell populations. Environ Sci Technol. 2022;56(4):2497–510. 35107992 10.1021/acs.est.1c07510

[25] Wang L, Zhang H, Song L, Guo X. Loss of allele diversity in introduced populations of the hermaphroditic bay scallop Argopecten irradians. Aquaculture. 2007;271(1–4):252–9.

[26] Fedele L, Brand T. The intrinsic cardiac nervous system and its role in cardiac pacemaking and conduction. J Cardiovasc Dev Dis. 2020;7(4):54. 33255284 10.3390/jcdd7040054 PMC7712215

[27] Lu M, Hayat R, Zhang X, Jiao Y, Huang J, Huangfu Y, et al. Comparative analysis of the cardiac structure and transcriptome of scallop and snail, perspectives on heart chamber evolution. Mar Life Sci Technol. 2023;5(4):478–91. 38045548 10.1007/s42995-023-00202-0 PMC10689705

[28] Filomeni G, De Zio D, Cecconi F. Oxidative stress and autophagy: the clash between damage and metabolic needs. Cell Death Differ. 2015;22(3):377–88. 25257172 10.1038/cdd.2014.150 PMC4326572

[29] Li A, Gao M, Liu B, Qin Y, Chen L, Liu H, et al. Mitochondrial autophagy: molecular mechanisms and implications for cardiovascular disease. Cell Death Dis. 2022;13(5):444. 35534453 10.1038/s41419-022-04906-6 PMC9085840

[30] Swirski FK, Nahrendorf M. Cardioimmunology: the immune system in cardiac homeostasis and disease. Nat Rev Immunol. 2018;18(12):733–44. 30228378 10.1038/s41577-018-0065-8

[31] Furtado MB, Nim HT, Boyd SE, Rosenthal NA. View from the heart: cardiac fibroblasts in development, scarring and regeneration. Development. 2016;143(3):387–97. 26839342 10.1242/dev.120576

[32] Hall C, Gehmlich K, Denning C, Pavlovic D. Complex relationship between cardiac fibroblasts and cardiomyocytes in health and disease. J Am Heart Assoc. 2021;10(5):e019338. 33586463 10.1161/JAHA.120.019338 PMC8174279

[33] D’Oria R, Schipani R, Leonardini A, Natalicchio A, Perrini S, Cignarelli A, et al. The role of oxidative stress in cardiac disease: from physiological response to injury factor. Oxid Med Cell Longev. 2020;2020(1):5732956. 32509147 10.1155/2020/5732956 PMC7244977

[34] Fukuda K. Development of regenerative cardiomyocytes from mesenchymal stem cells for cardiovascular tissue engineering. Artif Organs. 2001;25(3):187–93. 11284885 10.1046/j.1525-1594.2001.025003187.x

[35] Pinnamaneni JP, Singh VP, Kim MB, Ryan CT, Pugazenthi A, Sanagasetti D, et al. p63 silencing induces epigenetic modulation to enhance human cardiac fibroblast to cardiomyocyte-like differentiation. Sci Rep. 2022;12(1):11416. 35794145 10.1038/s41598-022-15559-y PMC9259667

[36] Galdos FX, Guo Y, Paige SL, VanDusen NJ, Wu SM, Pu WT. Cardiac regeneration: lessons from development. Circ Res. 2017;120(6):941–59. 28302741 10.1161/CIRCRESAHA.116.309040 PMC5358810

[37] Bateman A, Sandford R. The PLAT domain: a new piece in the PKD1 puzzle. Curr Biol. 1999;9(16):S1–2. 10.1016/s0960-9822(99)80380-7 10469604

[38] Kienesberger PC, Pulinilkunnil T, Nagendran J, Dyck JR. Myocardial triacylglycerol metabolism. J Mol Cell Cardiol. 2013;55:101–10. 22789525 10.1016/j.yjmcc.2012.06.018

[39] Menazza S, Blaauw B, Tiepolo T, Toniolo L, Braghetta P, Spolaore B, et al. Oxidative stress by monoamine oxidases is causally involved in myofiber damage in muscular dystrophy. Hum Mol Genet. 2010;19(21):4207–15. 20716577 10.1093/hmg/ddq339

[40] Lopaschuk GD, Karwi QG, Tian R, Wende AR, Abel ED. Cardiac energy metabolism in heart failure. Circ Res. 2021;128(10):1487–513. 33983836 10.1161/CIRCRESAHA.121.318241 PMC8136750

[41] Xu F, Gao T, Liu X. Metabolomics adaptation of juvenile pacific abalone Haliotis discus hannai to heat stress. Sci Rep. 2020;10(1):6353. 32286374 10.1038/s41598-020-63122-4 PMC7156721

[42] Tomanek L, Zuzow MJ. The proteomic response of the mussel congeners Mytilus galloprovincialis and M. trossulus to acute heat stress: implications for thermal tolerance limits and metabolic costs of thermal stress. J Exp Biol. 2010;213(20):3559–74. 20889836 10.1242/jeb.041228

[43] Dong YW, Liao ML, Han GD, Somero GN. An integrated, multi-level analysis of thermal effects on intertidal molluscs for understanding species distribution patterns. Biol Rev. 2022;97(2):554–81. 34713568 10.1111/brv.12811

[44] Bridge JH, Spitzer KW, Ershler PR. Relaxation of isolated ventricular cardiomyocytes by a voltage-dependent process. Science. 1988;241(4867):823–5. 3406740 10.1126/science.3406740

[45] Moreira LM, Takawale A, Hulsurkar M, Menassa DA, Antanaviciute A, Lahiri SK, et al. Paracrine signalling by cardiac calcitonin controls atrial fibrogenesis and arrhythmia. Nature. 2020;587(7834):460–5. 33149301 10.1038/s41586-020-2890-8

[46] Xie D, Xiong K, Su X, Wang G, Ji Q, Zou Q, et al. Identification of an endogenous glutamatergic transmitter system controlling excitability and conductivity of atrial cardiomyocytes. Cell Res. 2021;31(9):951–64. 33824424 10.1038/s41422-021-00499-5 PMC8410866

[47] Casselman M, Anttila K, Farrell A. Using maximum heart rate as a rapid screening tool to determine optimum temperature for aerobic scope in Pacific salmon Oncorhynchus spp. J Fish Biol. 2012;80(2):358–77. 22268435 10.1111/j.1095-8649.2011.03182.x

[48] Pörtner HO, Knust R. Climate change affects marine fishes through the oxygen limitation of thermal tolerance. Science. 2007;315(5808):95–7. 17204649 10.1126/science.1135471

[49] Zhu G, Fang Q, Zhu F, Huang D, Yang C. Structure and function of pancreatic lipase-related protein 2 and its relationship with pathological states. Front Genet. 2021;12:693538. 34290745 10.3389/fgene.2021.693538 PMC8287333

[50] De Caro J, Sias B, Grandval P, Ferrato F, Halimi H, Carrière F, et al. Characterization of pancreatic lipase-related protein 2 isolated from human pancreatic juice. Biochim Biophys Acta Proteins Proteom. 2004;1701(1–2):89–99. 10.1016/j.bbapap.2004.06.005 15450178

[51] Tao HP, Lu TF, Li S, Jia GX, Zhang XN, Yang QE, et al. Pancreatic lipase-related protein 2 is selectively expressed by peritubular myoid cells in the murine testis and sustains long-term spermatogenesis. Cell Mol Life Sci. 2023;80(8):217. 37468762 10.1007/s00018-023-04872-y PMC11072130

[52] Xu L, Huang HJ, Zhou X, Liu CW, Bao YY. Pancreatic lipase-related protein 2 is essential for egg hatching in the brown planthopper. Nilaparvata lugens Insect Mol Biol. 2017;26(3):277–85. 28032922 10.1111/imb.12290

[53] Agrawal N, Dasaradhi P, Mohmmed A, Malhotra P, Bhatnagar RK, Mukherjee SK. RNA interference: biology, mechanism, and applications. Microbiol Mol Biol Rev. 2003;67(4):657–85. 14665679 10.1128/MMBR.67.4.657-685.2003 PMC309050

[54] Lowe ME. Properties and function of pancreatic lipase related protein 2. Biochimie. 2000;82(11):997–1004. 11099796 10.1016/s0300-9084(00)01184-6

[55] Listenberger LL, Han X, Lewis SE, Cases S, Farese RV Jr, Ory DS, et al. Triglyceride accumulation protects against fatty acid-induced lipotoxicity. Proc Natl Acad Sci U S A. 2003;100(6):3077–82. 12629214 10.1073/pnas.0630588100 PMC152249

[56] Masanja F, Yang K, Xu Y, He G, Liu X, Xu X, et al. Impacts of marine heat extremes on bivalves. Front Mar Sci. 2023;10:1159261.

[57] Ahmed-Farid OA, Salah AS, Nassan MA, El-Tarabany MS. Effects of chronic thermal stress on performance, energy metabolism, antioxidant activity, brain serotonin, and blood biochemical indices of broiler chickens. Animals. 2021;11(9):2554. 34573520 10.3390/ani11092554 PMC8467978

[58] Yu Y, Tong D, Yu Y, Tian D, Zhou W, Zhang X, et al. Toxic effects of four emerging pollutants on cardiac performance and associated physiological parameters of the thick-shell mussel (Mytilus coruscus). Environ Pollut. 2023;334:122244. 37482340 10.1016/j.envpol.2023.122244

[59] Chuang SJ, Johanns M, PyrditRuys S, Steinberg GR, Kemp BE, Viollet B, et al. AMPK activation by SC4 inhibits noradrenaline-induced lipolysis and insulin-stimulated lipogenesis in white adipose tissue. Biochem J. 2021;478(21):3869–89. 34668531 10.1042/BCJ20210411

[60] Hong J, Park E, Lee J, Lee Y, Rooney BV, Park Y. Exercise training mitigates ER stress and UCP2 deficiency-associated coronary vascular dysfunction in atherosclerosis. Sci Rep. 2021;11(1):15449. 34326395 10.1038/s41598-021-94944-5 PMC8322067

[61] Attaallah A, Marchionni S, El-Beltagy A, Abdelaziz K, Lorenzini A, Milani L. Cell cultures of the Manila clam and their possible use in biomonitoring and species preservation. Eur Zool J. 2020;87(1):624–41.

[62] Curtis T, Depledge M, Williamson R. Voltage-activated currents in cardiac myocytes of the blue mussel, Mytilus edulis. Comp Biochem Physiol A Mol Integr Physiol. 1999;124(2):231–41.

[63] Little TJ, Hultmark D, Read AF. Invertebrate immunity and the limits of mechanistic immunology. Nat Immunol. 2005;6(7):651–4. 15970937 10.1038/ni1219

[64] Wang X, Wang L, Zhang H, Ji Q, Song L, Qiu L, et al. Immune response and energy metabolism of Chlamys farreri under Vibrio anguillarum challenge and high temperature exposure. Fish Shellfish Immunol. 2012;33(4):1016–26. 22960216 10.1016/j.fsi.2012.08.026

[65] Schröder M, Kaufman RJ. ER stress and the unfolded protein response. Mutat Res. 2005;569(1–2):29–63. 15603751 10.1016/j.mrfmmm.2004.06.056

[66] Lucas A, Mialet-Perez J, Daviaud D, Parini A, Marber MS, Sicard P. Gadd45 γ regulates cardiomyocyte death and post-myocardial infarction left ventricular remodelling. Cardiovasc Res. 2015;108(2):254–67. 26370247 10.1093/cvr/cvv219

[67] Kim MY, Seo EJ, Lee DH, Kim EJ, Kim HS, Cho HY, et al. Gadd45β is a novel mediator of cardiomyocyte apoptosis induced by ischaemia/hypoxia. Cardiovasc Res. 2010;87(1):119–26. 20154065 10.1093/cvr/cvq048

[68] Yazawa M, Ferrante C, Feng J, Mio K, Ogura T, Zhang M, et al. TRIC channels are essential for Ca2+ handling in intracellular stores. Nature. 2007;448(7149):78–82. 17611541 10.1038/nature05928

[69] Guerrero-Juarez CF, Dedhia PH, Jin S, Ruiz-Vega R, Ma D, Liu Y, et al. Single-cell analysis reveals fibroblast heterogeneity and myeloid-derived adipocyte progenitors in murine skin wounds. Nat Commun. 2019;10(1):650. 30737373 10.1038/s41467-018-08247-x PMC6368572

[70] Deb A, Ubil E. Cardiac fibroblast in development and wound healing. J Mol Cell Cardiol. 2014;70:47–55. 24625635 10.1016/j.yjmcc.2014.02.017 PMC4028446

[71] Zhou P, Pu WT. Recounting cardiac cellular composition. Circ Res. 2016;118:368–70. 26846633 10.1161/CIRCRESAHA.116.308139 PMC4755297

[72] Segers VF, Brutsaert DL, De Keulenaer GW. Cardiac remodeling: endothelial cells have more to say than just NO. Front Physiol. 2018;9:382. 29695980 10.3389/fphys.2018.00382 PMC5904256

[73] Maeda-Martinez AN, Sicard MT, Reynoso-Granados T. A shipment method for scallop seed. J Shellfish Res. 2000;19(2):765–70.

[74] Dong YW, Williams GA. Variations in cardiac performance and heat shock protein expression to thermal stress in two differently zoned limpets on a tropical rocky shore. Mar Biol. 2011;158:1223–31.

[75] Xing Q, Zhang L, Li Y, Zhu X, Li Y, Guo H, et al. Development of novel cardiac indices and assessment of factors affecting cardiac activity in a bivalve mollusc Chlamys farreri. Front Physiol. 2019;10:293. 30967793 10.3389/fphys.2019.00293 PMC6438923

[76] Zhang A, Liu Y, Pan J, Pontanari F, Chang ACH, Wang H, et al. Delivery of mitochondria confers cardioprotection through mitochondria replenishment and metabolic compliance. Mol Ther. 2023;31(5):1468–79. 36805084 10.1016/j.ymthe.2023.02.016 PMC10188643

[77] Hu Y, Li XC, Wang ZH, Luo Y, Zhang X, Liu XP, et al. Tau accumulation impairs mitophagy via increasing mitochondrial membrane potential and reducing mitochondrial Parkin. Oncotarget. 2016;7(14):17356. 26943044 10.18632/oncotarget.7861 PMC4951217

[78] Dobin A, Davis CA, Schlesinger F, Drenkow J, Zaleski C, Jha S, et al. STAR: ultrafast universal RNA-seq aligner. Bioinformatics. 2013;29(1):15–21. 23104886 10.1093/bioinformatics/bts635 PMC3530905

[79] Stuart T, Butler A, Hoffman P, Hafemeister C, Papalexi E, Mauck WM, et al. Comprehensive integration of single-cell data. Cell. 2019;177(7):1888-902. e21. 31178118 10.1016/j.cell.2019.05.031 PMC6687398

[80] Cao J, Spielmann M, Qiu X, Huang X, Ibrahim DM, Hill AJ, et al. The single-cell transcriptional landscape of mammalian organogenesis. Nature. 2019;566(7745):496–502. 30787437 10.1038/s41586-019-0969-x PMC6434952

[81] Efremova M, Vento-Tormo M, Teichmann SA, Vento-Tormo R. Cell PhoneDB: inferring cell–cell communication from combined expression of multi-subunit ligand–receptor complexes. Nat Protoc. 2020;15(4):1484–506. 32103204 10.1038/s41596-020-0292-x

[82] Zhu X, Liao H, Yang Z, Peng C, Lu W, Xing Q, et al. Genome-wide identification, characterization of RLR genes in Yesso scallop (Patinopecten yessoensis) and functional regulations in responses to ocean acidification. Fish Shellfish Immunol. 2020;98:488–98. 31978530 10.1016/j.fsi.2020.01.036

[83] Kumar S, Stecher G, Tamura K. MEGA7: molecular evolutionary genetics analysis version 7.0 for bigger datasets. Mol Biol Evol. 2016;33(7):1870–4. 27004904 10.1093/molbev/msw054 PMC8210823

[84] Kassambara A. ggpubr:'ggplot2'based publication ready plots. R package version 0.6.0. 2023. https://rpkgs.datanovia.com/ggpubr/.

[85] Wang D, Liu N, Kong X, Zhu X, Wang Y, Hu J, et al. Single-cell transcriptomic dynamics of scallop heart reveals the heterogeneous response to heat stress. https://www.ncbi.nlm.nih.gov/sra/?term=PRJNA1187280. 10.1186/s12915-025-02210-1 PMC12001498 40234911

