# Supplementary material for: Single-cell transcriptomic dynamics of scallop heart reveals the heterogeneous response to heat stress
Source: BMC Biol. 2025 Apr 15;23:98. doi: 10.1186/s12915-025-02210-1 (PMC12001498; doi:10.1186/s12915-025-02210-1)
Supplement: Supplementary file 1 — Additional file 1: Fig. S1. The histogram of heart rate of bay scallop in groups. Fig. S2. Expression profile of cardiac markers to categorize ventricle and atria in bay scallop. Fig. S3. Venn diagram and KEGG analysis of DEGs in five main cell types. Fig. S4. KEGG analysis of DEGs in CMs and ECs. Fig. S5. KEGG analysis of DEGs in FCs and ILCs. Fig. S6. The raw data of flow cytometry results from MTG and JC-1 analysis. Fig. S7. KEGG analysis of up- and down-regulated DEGs in six cell types. Fig. S8. Pseudotime ordering of cells, including AMs (cluster 12), VMs (cluster 6), and CPCs (cluster 9) in three groups. Fig. S9. The expression profile of PLRP2s(-likes) in representative vertebrates (human and mouse) and invertebrates (scallop and snail). Fig. S10. Fluorescence microscopy of pEGFP-C1-transfected cells and triglyceride levels across experimental groups. Fig. S11. The expression profile of DEGs in “Protein processing in the endoplasmic reticulum” pathway. [file 12915_2025_2210_MOESM1_ESM.docx]

**Supplementary figures**

**Figure S1**. The histogram of heart rate of bay scallop in groups. Microscopic examination (ME), including SEM and TEM analysis. Mitochondrial analysis (MTA), including MTG and JC-1 evaluation. Physiological and biochemical index (PBI), including in vivo content of three neurotransmitters, ATP, ROS, and energy-related (e.g., PK. PDH, LDH) enzyme activities. The SC represents the single-cell transcriptome analysis.

**Figure S2.**  Expression profile of cardiac markers to categorize ventricle and atria in bay scallop.

**Figure S3.** (A) Venn diagram of DEGs at different stress time points. (B) KEGG analysis of DEGs in five main cell types, displaying the significant terms (*P* < 0.01).

**Figure S4.** KEGG analysis of DEGs in (A) two clusters of cardiomyocytes (CMs) and (B) three clusters of endothelial cells (ECs), displaying the significant terms (*P* < 0.05).

**Figure S5.** KEGG analysis of DEGs in (A) two clusters of fibroblast cells (FCs) and (B) five clusters of immune-like cells (ILCs), displaying the significant terms (*P* < 0.05).

**Figure S6.** The raw data of flow cytometry results from MTG (A) and JC-1 (B) analysis.

**Figure S7.** KEGG analysis of up- and down-regulated DEGs in six cell types, displaying the significant terms (*P* < 0.05).

**Figure S8.**  Pseudotime ordering of cells, including AMs (cluster 12), VMs (cluster 6), and CPCs (cluster 9) in three groups. Lighter shades represent later pseudotime.

**Figure S9.** The expression profile of *PLRP2s*(-*likes*) in representative vertebrates (human and mouse) and invertebrates (scallop and snail).

**Figure S10.**  (A) The successfully transfected overexpressing plasmid pEGFP-C1 exhibited green under fluorescence scope. (B) The level of triglyceride content in different group cells.

**
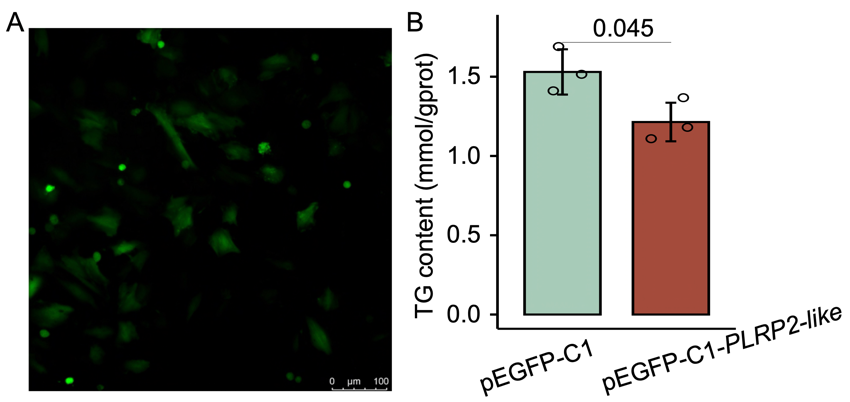
**

**Figure S11.**  The expression profile of DEGs in “Protein processing in the endoplasmic reticulum” pathway.
